# A metabolome atlas of mouse brain on the global metabolic signature dynamics following short-term fasting

**DOI:** 10.1038/s41392-023-01552-y

**Published:** 2023-09-08

**Authors:** Yaping Shao, Zhenfa Fu, Yanfeng Wang, Zhaofei Yang, Yushan Lin, Song Li, Cheng Cheng, Min Wei, Zheyi Liu, Guowang Xu, Weidong Le

**Affiliations:** 1 Liaoning Provincial Key Laboratory for Research on the Pathogenic Mechanisms of Neurological Diseases, The First Affiliated Hospital, Dalian Medical University, 193 Lianhe Road, 116021 Dalian, China; 2 CAS Key Laboratory of Separation Science for Analytical Chemistry, Dalian Institute of Chemical Physics, Chinese Academy of Sciences, 457 Zhongshan Road, 116023 Dalian, China; 3 Institute of Neurology, Sichuan Academy of Medical Science-Sichuan Provincial Hospital, Medical School of UESTC, 611731 Chengdu, China

**Keywords:** Systems biology, Neuroscience, Pathogenesis

## Abstract

Calorie restriction (CR) or a fasting regimen is considered one of the most potent non-pharmacological interventions to prevent chronic metabolic disorders, ameliorate autoimmune diseases, and attenuate aging. Despite efforts, the mechanisms by which CR improves health, particularly brain health, are still not fully understood. Metabolic homeostasis is vital for brain function, and a detailed metabolome atlas of the brain is essential for understanding the networks connecting different brain regions. Herein, we applied gas chromatography-mass spectrometry-based metabolomics and lipidomics, covering 797 structurally annotated metabolites, to investigate the metabolome of seven brain regions in fasted (3, 6, 12, and 24 h) and *ad libitum* fed mice. Using multivariate and univariate statistical techniques, we generated a metabolome atlas of mouse brain on the global metabolic signature dynamics across multiple brain regions following short-term fasting (STF). Significant metabolic differences across brain regions along with STF-triggered region-dependent metabolic remodeling were identified. We found that STF elicited triacylglycerol degradation and lipolysis to compensate for energy demand under fasting conditions. Besides, changes in amino acid profiles were observed, which may play crucial roles in the regulation of energy metabolism, neurotransmitter signaling, and anti-inflammatory and antioxidant in response to STF. Additionally, this study reported, for the first time, that STF triggers a significant elevation of *N*-acylethanolamines, a class of neuroprotective lipids, in the brain and liver. These findings provide novel insights into the molecular basis and mechanisms of CR and offer a comprehensive resource for further investigation.

## Introduction

With changes in modern lifestyles and the aggravation of the global aging population, the prevalence of chronic diseases including diabetes, cardiovascular disease, and age-related neurodegenerative disorders, has increased remarkably. Therefore, the prevention of disease and the preservation of good health have received special attention from the scientific and medical communities. Calorie restriction (CR) is considered one of the most potent and robust non-pharmacological interventions for attenuating aging and supporting metabolic health.^1,2^ CR forms can range from a chronic but mild reduction in calorie intake to intermittent periods of repeated cycles of short-term fasting (STF). Accumulating studies have confirmed that these various forms of CR have beneficial effects on health, including weight loss, amelioration of metabolic syndrome and cardiovascular disease, and lifespan extension.^3,4^ Clinical studies have also shown the benefits of CR in brain diseases, such as epilepsy, Alzheimer’s disease (AD), and multiple sclerosis.^5^ However, the mechanisms by which CR improves health, especially brain health, remain elusive.

The brain is characterized by a unique morphology of anatomical regions, each of which involves an enormous diversity of cell types and highly integrated molecular programs during brain development and functional maintenance.^6^ Metabolic homeostasis in the brain is essential for brain function. A detailed metabolome atlas of the brain is critical for understanding the networks connecting different brain regions. Clear regional differences in both brain metabolome and proteome have been unveiled recently.^6–9^ It has been revealed that, although glucose is the main energy supplier for the brain, different cell populations use glucose via distinct metabolic pathways, either by glycolysis or the pentose phosphate pathway (PPP).^8^ The mammalian brain relies on diverse small molecular metabolites such as neurotransmitters, lipids, and amino acids to fulfill its functions. Metabolites refer to the ultimate outcome of molecular biology, the profiles of which cannot be simply predicted by genomic, transcriptomic, or proteomic signatures owing to the multitude of feedback mechanisms and regulatory loops.^6^ Thus, a comprehensive analysis of metabolic reprogramming in response to CR may result in effective therapeutic strategies to prevent brain diseases and expand the health span.

In mammals, disturbance of normal diet patterns alters metabolism systemically.^4^ Previous work has indicated that changes in the cerebellum metabolites upon graded CR are related to feeding regulation.^10^ Increased insulin levels and upregulation of glucose transports in neurons and endothelial cells in hypothalamus have been found in rats during STF.^11^ Moreover, it has been reported that CR diets improve the aging-related dysregulation of brain regional gamma-aminobutyric acid (GABA) system, corticosterone status, and cognitive function.^12^ Nevertheless, the impact of CR or STF on spatiotemporal brain metabolome has been studied inadequately, only concentrating on limited metabolites or restricting to a few anatomical brain regions.^7,8^

Using gas chromatography-mass spectrometry (GC-MS)-based metabolomics and liquid chromatography-mass spectrometry (LC-MS)-based lipidomics techniques, the present study profiled the metabolic signatures of seven brain regions and delineated metabolic remodeling across different brain regions following various periods of fasting. We identified a series of metabolites that encompass diverse metabolic modules ranging from saccharides, lipids, amino acids, and nucleosides to amino acid neurotransmitters. Furthermore, this study investigated the metabolic changes in the brain in response to STF and uncovered remarkable remodeling of lipid and amino acid metabolism under conditions of food deprivation. By creating a metabolome atlas of the mouse brain based on the global metabolic signature dynamics across multiple brain regions during STF, we aimed to provide novel insights into the underlying mechanisms by which CR improves brain health and offer a comprehensive resource for future studies.

## Results

### Mouse brain metabolome coverage and data quality assessment

**Fig. 1:**
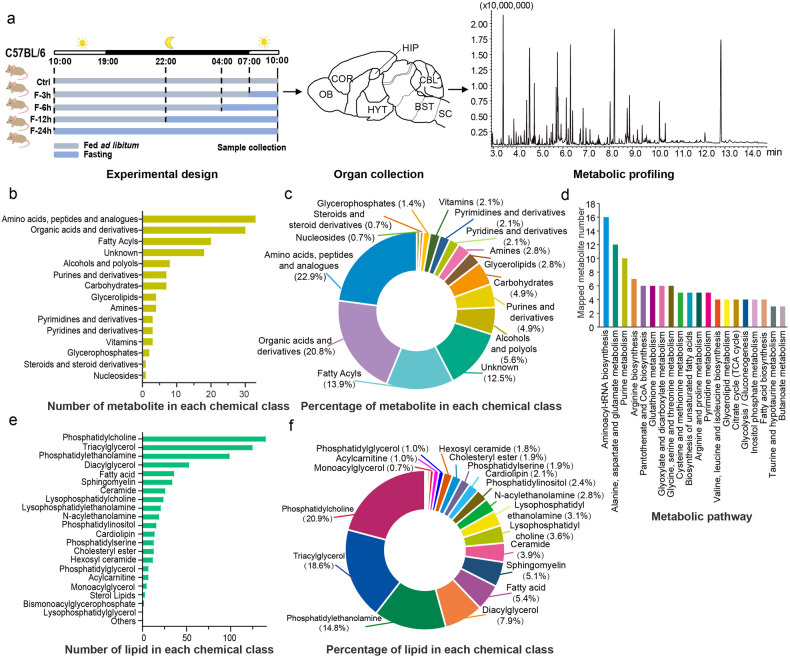
Overview of the mouse brain metabolome dataset. **a** Graphic illustration of the experimental design to acquire mouse brain metabolome dataset. **b** Number of the detected metabolites in each chemical class using HMDB classification scheme (GC-MS). **c** Percentage of each chemical class in the metabolites detected by GC-MS. **d** Top-20 mapped metabolic pathways of the annotated metabolites in GC-MS. **e** Number of the detected lipids in each chemical class. **f** Percentage of each lipid class

To depict the brain metabolome atlas across distinct regions and investigate the impact of the feeding regimen on global metabolic remodeling, we performed GC-MS-based metabolomics and LC-MS-based lipidomics on seven brain regions of C57BL/6 female mice subjected to different periods of fasting (3, 6, 12, and 24 h) or fed *ad libitum* (Fig. 1a). The seven anatomically defined regions included the olfactory bulb (OB), frontal cortex (COR), hypothalamus (HYT), hippocampus (HIP), cerebellum (CBL), brainstem (BST) and spinal cord (SC). Sagittal views of these brain regions are shown in Fig. 1a.^6^ For GC-MS analysis, using retention time, retention index, and mass spectral information from the NIST11 library and in-house database, the chromatographic peaks of 144 ion features were manually integrated, and 126 of them were annotated with definite structures (Supplementary Data 1). These metabolites were defined into 14 chemical classes according to the HMDB classification scheme. The number and percentage of metabolites in each chemical category are shown in Fig. 1b, c. These metabolites were mapped into 54 metabolic pathways using MetaboAnalyst 5.0 (https://www.metaboanalyst.ca, Fig. 1d).^13^ For lipidomics analysis, 801 lipid ion features in positive ionization mode and 431 lipid ion features in negative ionization mode were identified using MS-DIAL (ver. 4.93) software.^14^ After removing the lipids detected repeatedly by two ionization modes and those with low signal-to-noise ratio and high coefficient of variation, 671 unique lipid species of 22 lipid classes were retained (Supplementary Data 2, Fig. 1e, f). These data illustrate the adequate coverage of pathway modules in this study for in-depth profiling of complex brain regions.

Quality control (QC) samples were constructed by pipetting equal aliquots of brain extracts from all tissues and analyzed along with the samples to monitor signal fluctuation and reproducibility during the analytical process. To visualize the variation in the dataset, principal component analysis (PCA) in two-dimensional and three-dimensional score plots were performed. As shown in Supplementary Fig. 1a–e, the QC samples clustered tightly, demonstrating minimal residual technical errors. Using univariate analysis of the detected metabolites in QC samples, 92.1% and 89.7% of the annotated metabolites and lipids, respectively, showed excellent biological reproducibility with relative standard deviations of <30% in GC-MS and lipidomic analyses, suggesting that the data we acquired were robust and dependable (Supplementary Fig. 1c, f).

### Metabolic differences across brain regions

**Fig. 2:**
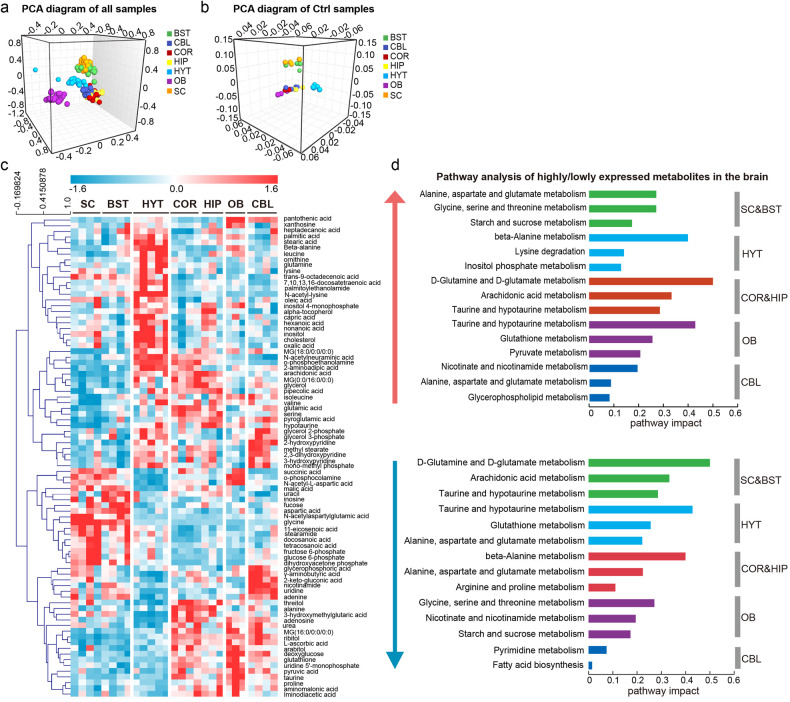
Metabolic profiles across various brain regions. **a** Three-dimensional diagram of PCA of brain metabolome from mice fed *ad libitum* or subjected to STF. R2X = 0.614, Q2 = 0.420 (GC-MS data). **b** Three-dimensional diagram of PCA of brain metabolome from mice fed *ad libitum*. R2X = 0.869, Q2 = 0.599 (GC-MS data). **c** Heat map of the differentially expressed metabolites (detected by GC-MS) among seven brain regions in mice fed *ad libitum*. **d** Pathway analysis of the highly expressed and lowly expressed metabolites in different brain regions. Pathway enrichment analysis was performed utilizing the Pathway Analysis module from online MetaboAnalyst 5.0 (https://www.metaboanalyst.ca/)

Since regional specialization is a hallmark of the architecture and function of the brain, we first investigated the differences in the metabolome across brain regions. To begin, we performed PCA on the metabolites identified across all brain regions and found segregation primarily according to the region type (Fig. 2a and Supplementary Fig. 2a). The metabolic profiles of the seven brain regions were integrated into five clusters. One cluster comprised BST and SC, which contain more axons than neuronal cell bodies and dendrites on the cellular component.^15^ One cluster comprised HIP and COR, which are enriched with neuronal cell bodies and dendrites and depleted in axons.^15^ As judged by PCA, the remaining three regions (OB, CBL, and HYT) exhibited three distinct metabolic profiles. PCA of samples from mice fed *ad libitum* also resulted in similar sample clustering (Fig. 2b and Supplementary Fig. 2b).

To identify the differentially expressed metabolites (DEMs) among the different brain regions, we performed a one-way analysis of variance (ANOVA) and identified 538 DEMs across the seven brain regions. Consistent with the PCA results, the variation in these DEMs was clustered into five patterns (Fig. 2c and Supplementary Fig. 2–5). Notably, we did not find any differences in the levels of glucose, fructose, or lactate across the brain regions. To better visualize the differences in metabolic pathways, we performed pathway enrichment analysis using metabolites with high or low expression in each brain region (Fig. 2d). For instance, the most abundant metabolites in the OB engaged in taurine and hypotaurine metabolism and glutathione metabolism, whereas low-abundance metabolites were involved in glycine, serine and threonine metabolism and nicotinate and nicotinamide metabolism. However, in HYT, the most abundant metabolites were implicated in the pathways of beta-alanine metabolism and lysine degradation, whereas metabolites in the pathways of taurine and hypotaurine metabolism, and glutathione metabolism were expressed at low levels. In addition, different brain regions exhibited distinct lipid compositions. Hierarchical clustering showed that phosphatidylethanolamine (PE) O- and hexosyl ceramide (HexCer) were enriched in SC and BST. Conversely, the COR and HIP brain regions showed diametrically opposing lipid compositions. High levels of ceramide (Cer) and sphingomyelin (SM) and low levels of N-acylethanolamine (NAE) were observed in HYT (Supplementary Fig. 3, 5).

### Effects of STF on the metabolome of different brain regions

**Fig. 3:**
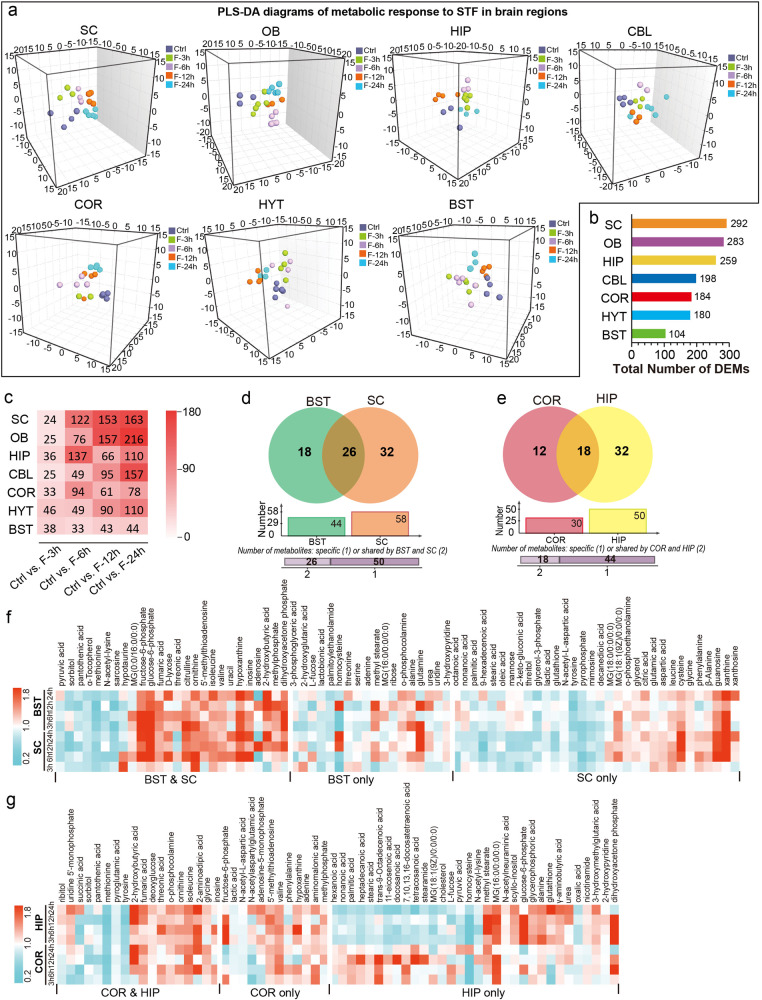
Spatiotemporal effects of STF on the metabolome in multiple brain regions. **a** Three-dimensional diagrams of PLS-DA of metabolic responses to different periods of STF in SC, R2X = 0.478, R2Y = 0.585, Q2 = 0.160; OB, R2X = 0.508, R2Y = 0.543, Q2 = 0.203; HIP, R2X = 0.497, R2Y = 0.603, Q2 = 0.0753; CBL, R2X = 0.426, R2Y = 0.562, Q2 = −0.0154; COR, R2X = 0.472, R2Y = 0.539, Q2 = 0.129; HYT, R2X = 0.522, R2Y = 0.497, Q2 = −0.00197 and BST, R2X = 0.472, R2Y = 0.522, Q2 = 0.0736. **b** Number of DEMs upon STF in seven brain regions. **c** Number of DEMs after various periods of STF in seven brain regions. Venn diagram of the number of DEMs (GC-MS data only) after STF in (**d**) BST and SC, (**e**) COR and HIP. Venn diagrams were generated using online tools (https://jvenn.toulouse.inrae.fr/app/example.html). More metabolites were found to be changed after STF in SC and HIP, compared with BST and COR, respectively. Heat maps of the DEMs (GC-MS data only) upon STF in (**f**) BST and SC, (**g**) COR and HIP

Next, we focused on the spatiotemporal effects of STF on brain metabolome. To achieve this, we performed partial least squares discriminant analysis (PLS-DA, Fig. 3a, Supplementary Fig. 6) and Student’s *t* test to identify significantly changed metabolites after different periods of fasting and found that the metabolic response to STF in the brain was region-dependent. By calculating the number of DEMs induced by STF in each brain region, we found that the metabolite components in the SC, OB, and HIP were the most affected, followed by CBL, COR, HYT, and BST (Fig. 3b). Our data also showed that the effect of STF on the brain metabolome was enhanced with the duration of fasting in the SC, OB, CBL, HYT, and BST. Conversely, the most profound effects on the metabolome of COR and HIP were observed after 6 h of fasting (Fig. 3c). We previously mentioned that similar metabolic phenotypes were identified between SC and BST, as well as between COR and HIP, and our results further indicated that the impact of STF duration was similar between SC and BST, as well as between COR and HIP. However, more profound metabolic alterations were observed in the SC and HIP than in the BST and COR during STF (Fig. 3d, e, Supplementary Fig. 7). Except for DEMs that were identified only in SC (or COR) or BST (or HIP), similar response patterns were found for DEMs identified in both SC and BST (or COR and HIP) during STF (Fig. 3f, g, Supplementary Data 3). Detailed information on the differentially expressed lipids and metabolites after each fasting period is provided in Supplementary Data 4, 5. The results showed that STF elicited broad metabolic remodeling of lipid and amino acid metabolism in the brain.

#### Remodeling of lipid metabolism

**Fig. 4:**
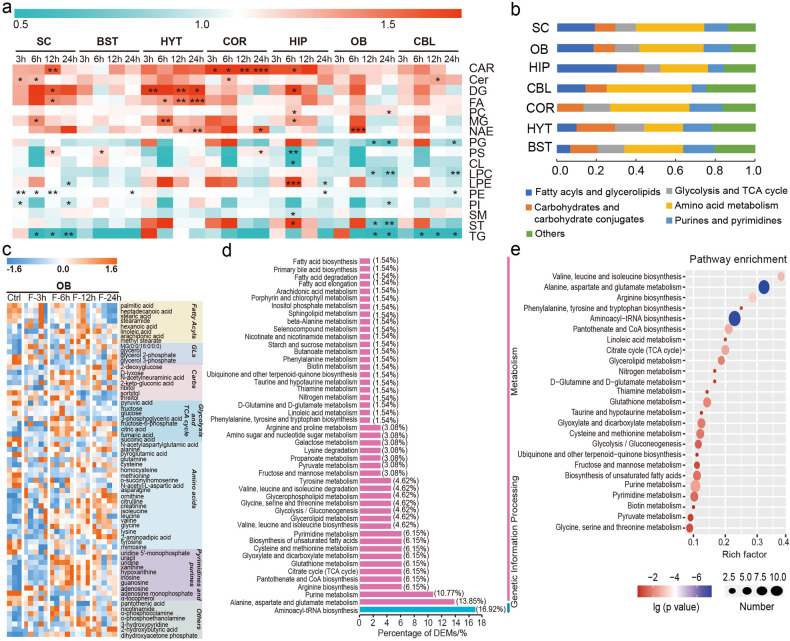
Visualization and pathway analysis of the differentially expressed lipids and metabolites that responded to fasting in the brain. **a** Change of total abundance of each lipid class after STF in seven brain regions. *0.01 < *p* < 0.05, **0.001 < *p* < 0.01, ***p < 0.001. CAR, acylcarnitine; Cer, ceramide; DG, diacylglycerol; FA, fatty acid; PC, phosphatidylcholine; MG, monoacylglycerol; NAE, N-acylethanolamine; PG, phosphatidylglycerol; PS, phosphatidylserine; CL, cardiolipin; LPC, lysophosphatidylcholine; LPE, lysophosphatidylethanolamine; PE, phosphatidylethanolamine; PI, phosphatidylinositol; SM, sphingomyelin; ST, sterol lipids; TG, triacylglycerol. **b** Summary of the categories of differential metabolites detected by GC-MS in seven brain regions. **c** Heat map of DEMs (GC-MS data) in OB after STF. **d** Percentage of DEMs (GC-MS data) in different metabolic pathways in OB. **e** Top-25 metabolic pathways (GC-MS data) that were associated with STF in OB. The rich factor indicates the ratio of the number of DEMs in the indicated metabolic pathway to the number of all metabolites annotated to this pathway, the higher the value, the higher the enrichment degree. The “Number” in circle size represents the number of DEMs that fall into the indicated pathway. Pathway enrichment analysis was performed utilizing the Pathway Analysis module from online MetaboAnalyst 5.0 (https://www.metaboanalyst.ca/)

STF induced marked alterations in the glycerolipid levels in all brain regions. The abundance of diacylglycerol (DG), monoacylglycerol (MG), and fatty acid (FA) increased, whereas total triacylglycerol (TG) levels declined after STF (Fig. 4a). Each lipid species in the MG, FA, and TG followed the same pattern, except for DG. DGs having a long chain containing a total number of carbon atoms greater than 50 decreased, while those containing total carbon atoms less than 50 increased after STF (Supplementary Data 4). STF also affected the levels of sphingolipids (Cer and SM) and phospholipids in the brain. However, the alteration of each lipid species in these lipid classes followed different patterns across brain regions. In addition, elevated levels of NAEs, a class of neuroprotective lipids, were noted after STF. These findings indicate remodeling of lipid composition in the brain during fasting.

Apart from lipid metabolism, a global alteration in amino acids was also observed during STF (Fig. 4b–e, Supplementary Fig. 8–13). Pathway enrichment analysis indicated that the biosynthesis of valine, leucine, and isoleucine (branched-chain amino acids, BCAAs) and phenylalanine, tyrosine, and tryptophan (aromatic amino acids, ArAAs), together with arginine, alanine, aspartate, and glutamate metabolism, were the most representative pathways related to STF (Fig. 4e, Supplementary Fig. 8–13).

#### Remodeling of amino acid metabolism

**Fig. 5:**
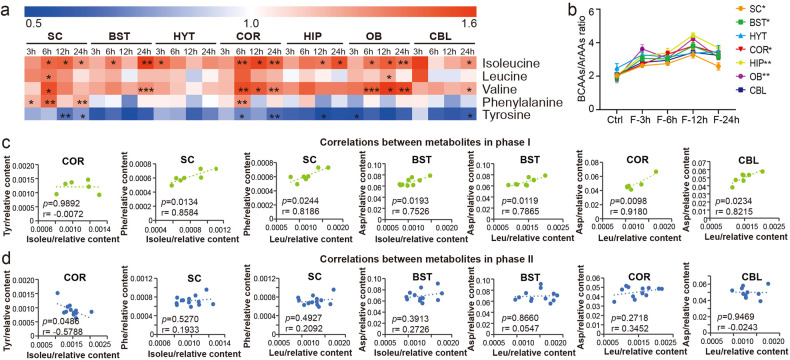
Changes of BCAAs and ArAAs in the brain during STF. **a** Heat map of BCAA and ArAA levels after different periods of fasting in seven brain regions. **b** Changes of BCAAs/ArAAs ratios after STF in seven brain regions. One-way ANOVA was used to identify differences between different time points in each tissue. **c** Correlation between metabolites in phase I. **d** Correlation between metabolites in phase II. Isoleu, isoleucine; Val, valine; Leu, leucine; Tyr, tyrosine; Phe, phenylalanine; Asp, aspartic acid. *0.01 < *p* < 0.05, **0.001 < *p* < 0.01, ***p < 0.001

First, we noted a global elevation in BCAAs levels across seven brain regions. In contrast, decreased level of tyrosine (SC, COR, HIP, OB, and CBL) and increased level of phenylalanine (SC, COR), both of which are ArAAs, were observed during STF (Fig. 5a). Moreover, the ratio of BCAAs/ArAAs markedly increased in the SC, BST, COR, HIP, and OB during fasting (Fig. 5b, Supplementary Data 6). Considering that fasting for 3 h had a slight effect on the levels of these metabolites, we divided the samples into two phases (phase I: Ctrl and F-3 h; phase II, F-6 h, F-12 h, and F-24 h) to investigate the correlation between metabolites in different fasting states. In the COR, a significant inverse correlation was found between the tyrosine and isoleucine levels after a longer period of fasting (Fig. 5c, d). However, positive phenylalanine/aspartate (Asp)-isoleucine/leucine correlations were disrupted after a longer fasting duration (Fig. 5d).

**Fig. 6:**
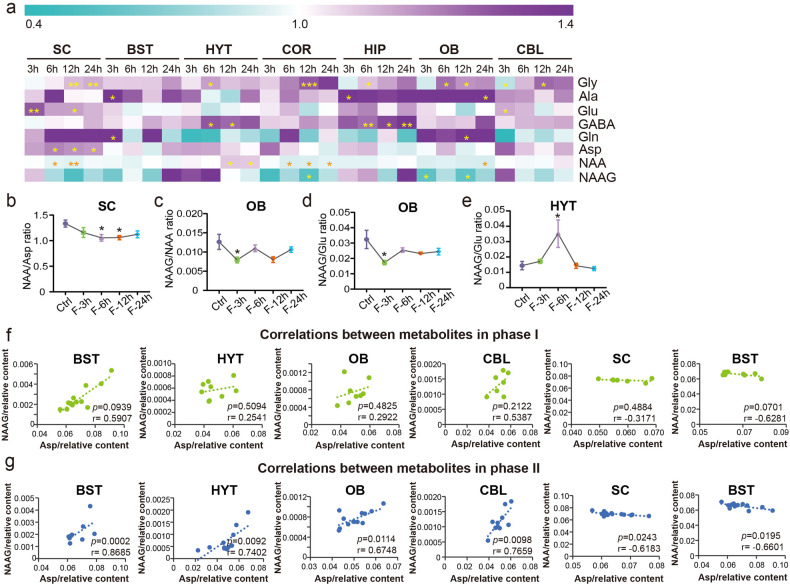
Changes of amino acid neurotransmitters in the brain during STF. **a** Heat map of the differentially expressed amino acid neurotransmitters upon STF in seven brain regions. **b** Change of NAA/Asp ratio after STF in SC. **c**, **d** Changes of NAAG/NAA and NAAG/Glu ratios after STF in OB. **e** Change of NAAG/Glu ratio after STF in HYT. **f** Correlation between metabolites in phase I. **g** Correlation between metabolites in phase II. Gly, glycine; Ala, alanine; Glu, glutamate; GABA, gamma-aminobutyric acid; Gln, glutamine; Asp, aspartic acid; NAA, N-acetyl-L-aspartic acid; NAAG, N-acetylaspartylglutamic acid. *0.01 < *p* < 0.05, **0.001 < *p* < 0.01, ***p < 0.001

BCAAs can provide nitrogen for the synthesis of glutamic acid (Glu) and GABA, both of which increased during STF (Fig. 6a). Excess of Glu can be neurotoxic and can be consumed through alanine aminotransferase by converting pyruvate to alanine. As a result, declined pyruvate and elevated alanine levels were noted in most brain regions during STF (Supplementary Data 5). To better understand the potential mechanism underlying the metabolic changes, we reanalyzed previously published RNA-sequencing data on transcriptomic changes across different brain regions during STF.^4^ Consistently, a significant increase in the mRNA expression of *Gpt2*, which encodes alanine aminotransferase, was noted in CBL and BST (Supplementary Data 7). However, levels of *N*-acetylaspartylglutamic acid (NAAG, COR, and OB) and N-acetyl-l-aspartic acid (NAA, SC, COR, and OB) decreased after fasting (Fig. 6a). In addition, the NAA/Asp ratio decreased in the SC, indicating a possible reduction in enzyme activity that catalyzes the synthesis of NAA from Asp during fasting (Fig. 6b). The NAAG/NAA and NAAG/Glu ratios were also reduced in the OB (Fig. 6c, d). To support this, the mRNA expression of *Rimkla* and *Rimklb*, which encode enzymes that convert NAA to NAAG, was significantly decreased in the OB after STF (Supplementary Data 7), which might indicate suppressed synthesis of NAAG during fasting. In contrast, the NAAG/Glu ratio was elevated in HYT after fasting for 6 h, which may have contributed to the elevation of NAA in HYT (Fig. 6e). In addition, our results showed that a longer fasting period could induce a positive NAAG-Asp correlation but an inverse NAA-Asp correlation in the brain (Fig. 6f, g).

In addition to the results mentioned above, elevated levels of citrulline (OB, SC and BST) and ornithine (OB, CBL, COR, HIP, SC and BST) and reduced levels of O-succinylhomoserine (OB), homocysteine (Hcy, OB, HIP, HYT and BST)) and methionine (seven brain regions) were also observed in the brain during STF (Supplementary Data 5). Due to the lack of ornithine carbamoilotransferase in the mouse brain, citrulline cannot be produced from ornithine normally.^16^ Alternatively, citrulline can be recycled via citrulline–nitric oxide (NO) cycle through argininosuccinate synthetase, argininosuccinate lyase and nitric oxide synthase.^16^ We found that the mRNA expression levels of *Ass1* (OB), *Asl* (CBL) and *Nos1* (OB and CBL) increased after fasting, which might indicate an activation of citrulline–NO cycle during STF (Supplementary Data 7).

### Distinct effects of STF on the brain and liver metabolome

**Fig. 7:**
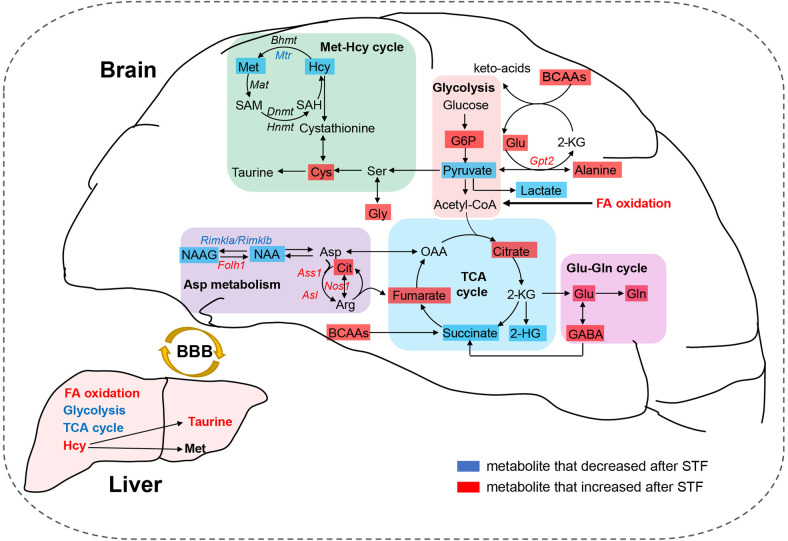
Metabolic remodeling in the brain and liver following STF. STF induced a global remodeling of lipid and amino acid metabolism, especially with respect to BCAA, ArAA, neurotransmitter, methionine and the citrulline–NO cycle for the adaptive responses of the brain under limited nutrition conditions. In contrast to the brain, STF induced significant remodeling of energy metabolism in the liver. Enhanced FA oxidation and declined glycolysis and TCA cycle, were found in liver in the fasted state. The metabolites and genes were linked to pathways using the Kyoto Encyclopedia of Genes and Genomes (KEGG) database (https://www.kegg.jp/kegg/pathway.html)

Given that the crosstalk between the liver and brain is crucial for maintaining glucose homeostasis during fasting, we investigated and compared the metabolic effects of STF in the brain and liver to identify potential network connections between these two organs. In total, 446 STF-related metabolites, including 376 lipids, were identified in the liver. As expected, increased acylglycerol and FA levels and decreased cholesteryl ester and phospholipid levels were determined during fasting (Supplementary Fig. 14a). Besides, elevated 3-hydroxybutyric acid and reduced levels of monosaccharides, disaccharides and trisaccharides were observed after 6 h of fasting (Supplementary Fig. 14b). The level of 3-hydroxybutyric acid positively correlated with the levels of FAs (Supplementary Fig. 15). In addition, decreased levels of malic acid and fumaric acid, two major metabolites of the tricarboxylic acid (TCA) cycle, were observed after 3 h of fasting. These results are consistent with those of previous studies demonstrating enhanced FA oxidation and reduced glucose production and utilization in the liver in the fasting state. However, we did not find any global alterations in amino acid levels in the liver after STF. In contrast to the brain, the level of Hcy markedly increased in the liver after STF. The remodeling of metabolic pathways during STF in the brain and liver is shown in Fig. 7.

To ascertain whether STF induces adaptive metabolic responses in the brain and liver, we investigated the lipidome of mice after 24 h of fasting and 24 h of refeeding (Ref group). The OPLS-DA score plot demonstrated that the Ref group remained distinguishable from the Ctrl group, indicating that the metabolic effects of fasting were not entirely reversed even after refeeding (Supplementary Fig. 6). The impact of refeeding on lipid species appeared to be multifaceted, with some lipid levels returning to the pre-fasted state, whereas others were further elevated or decreased (Supplementary Data 4, 8, Supplementary Fig. 16).

## Discussion

The impact of STF on brain health is complex and the underlying molecular mechanisms are poorly understood. Comprehensive mapping of the spatiotemporal molecular organization of the brain is essential to understand how the brain functions under specific conditions. In the current study, we performed both metabolomics and lipidomics to generate a metabolome atlas of the mouse brain, outlining global metabolic signature dynamics across seven brain regions following STF. This atlas represents the most comprehensive brain metabolome associated with STF published so far. Our results confirmed previous findings that the mouse brain metabolome is region-dependent and that metabolic differences across regions are primarily driven by anatomical similarities. Moreover, this study unveiled marked metabolic remodeling of lipids and amino acids in the brain during STF. These changes may be network-connected and play crucial roles in the adaptive response of the brain under fasting conditions. While recapitulating the reported molecular effects including enhanced lipolysis and FA oxidation and reduced glucose production and utilization in the liver during CR or fasting,^17,18^ this study reported for the first time that STF could induce the elevation of NAEs, a class of neuroprotective lipids, in both the brain and liver. These findings present a comprehensive resource on the region-specific metabolome across multiple brain regions and provide additional insights into new protective mechanisms of CR in the brain.

Remarkable metabolic differences were identified among the different brain regions (Fig. 2, Supplementary Fig. 2). Each brain region is enriched in a set of specific metabolites, which may be attributed to the distinct regional anatomy of the brain and the diverse cell types that make up each region, each with their unique function and metabolism.^8^ Myelin sheath is a multilayered membrane that plays an important role in insulating axons and rapid nerve conduction.^15^ The abundant myelin sheath in BST and SC leads to a significant enrichment of PE O-and HexCer, two lipid classes particularly enriched in myelin,^15^ in these tissues. Besides, high content of Asp and its peptide neurotransmitter derivatives (NAA and NAAG) in BST and SC may be beneficial to facilitate myelination by regulating histone H3 methylation in oligodendrocytes.^19,20^ The abundance of two principal neurotransmitters, Glu and GABA, was found to be associated with the distribution of glutamatergic and GABAergic neurons, showing that Glu and GABA are abundant in COR, HIP, and CBL, but lower in SC and HYT.^21^ Importantly, brain regions including COR, HIP, OB, and CBL also presented enriched antioxidants such as glutathione and L-ascorbic acid. These metabolic patterns may contribute to the implementation of learning, memory, and antioxidant capacities in these brain regions.

Furthermore, our results suggest that STF can trigger region-dependent metabolic responses across distinct brain regions, with metabolites and lipids in SC, OB, and HIP being the most significantly affected (Fig. 3, Supplementary Fig. 6). Nonetheless, common metabolic pathways involved in lipid and amino acid metabolism that are affected by STF were identified in all seven brain regions (Fig. 4, Supplementary Fig. 8-13). These results indicate that to some extent, the metabolic response of the brain to STF is achieved through biological perturbations shared among multiple brain regions. Crosstalk between the brain and other peripheral organs, such as the liver, has been shown in the context of fasting.^17,22^ During the fasting state, glucose homeostasis between the brain and liver is regulated through PPARα and FGF21 signaling.^22^ The enhanced lipolysis in adipose tissues results in the liberation of FAs and glycerol into the liver.^17^ The produced FAs can be oxidized via beta-oxidation in the liver and generate acetyl-CoA for hepatic gluconeogenesis using substrates of glycerol, lactate, and alanine.^17^ Meanwhile, the generated ketone bodies can be used as energy sources for both the body and the brain (Supplementary Fig. 14). These metabolic adaptations help maintain glucose homeostasis and ensure normal brain function. The present study further highlights the importance of STF-modulated metabolic networks among brain regions in adaptive responses during fasting.

Brain is the second most lipid-enriched organ after adipose tissues. Lipid homeostasis in the brain is closely related to energy metabolism, oxidative stress, synaptogenesis and neurogenesis, signaling transmission and neuroinflammation.^23^ We found that STF could elicit heightened TG degradation and lipolysis together with increased FAs in most brain regions (Fig. 4a). Significant transcriptional changes in genes involved in TG degradation have been reported in rodent HYT following short-term CR, supporting our findings at the transcriptome level.^24^ FAs are essential building blocks for membrane architecture and substrates for energy reserve. Due to the limited FA breakdown capacity of the brain, FAs are typically stored within cells as energy-rich TGs localized in lipid droplets (LDs).^25^ During periods of nutrient depletion, LDs can deliver FAs into astrocytic mitochondria, thus providing fuel for oxidative phosphorylation.^25^ It has been reported that STF induces profound neuronal autophagy.^25,26^ The FAs liberated by autophagy-mediated breakdown of membrane organelles can be packed and stored in new neuronal LDs, and then transported into the mitochondria of nearby astrocytes via ApoE-positive lipid particles.^25^ Therefore, the neuronal-derived FAs can be consumed thought beta-oxidation to reduce lipotoxicity and provide energy under fasted state. In addition, polyunsaturated FAs (PUFAs) and their biologically active derivatives have been shown to regulate various processes in the brain including neurotransmission, cell survival, neurogenesis, neuroinflammation, and synaptic function.^27^ Decreased levels of total PUFAs have been accompanied by aging and neurodegenerative diseases.^28^ Previous studies have shown that dietary supplementation with a specific ratio of n-3/n-6 PUFA can restore many age-related effects.^27,28^ Therefore, the favorable effects of STF or CR on brain health may be associated with increased lipolysis and elevated PUFAs exerted by fasting (Supplementary Data 4).

In addition to lipids, broad remodeling of amino acids, including BCAAs, ArAAs, neurotransmitters, the citrulline–NO cycle, and methionine, was also identified in the brain during STF. BCAAs are important nitrogen donors for the synthesis of Glu and GABA in the brain, and all these three amino acids can serve as substrates for energy metabolism by supplementing intermediate metabolites for the TCA cycle.^29^ Our results showed that STF could increase the levels of BCAAs, Glu, and GABA in the brain (Figs. 5a and 6a). Glu and GABA are extensively recycled between neurons and astrocytes via the Glu/GABA-glutamine cycle, which is vital for synaptic transmission and energy metabolism.^30^ Studies have shown that CR can modulate astrocyte functions and exert neuroprotective effects by increasing Glu uptake and glutamine synthetase activity in rats.^31^ Moreover, CR can augment the expression of Glu decarboxylase, thereby increasing GABA production.^32^ However, an excess of Glu can be neurotoxic. We found that STF may trigger an enhanced synthesis of alanine from pyruvate, which consumes Glu via alanine aminotransferase, thereby attenuating excitotoxicity in brain tissues.^33^ Conversely, NAA and NAAG levels declined in the brain during fasting, which is in line with the hypothesis that ketone bodies as fuel reduce Glu transamination to Asp.^33^ The breakdown of NAA and NAAG can produce acetyl groups and Asp that have the potential to modulate TCA cycle activity by affecting α-ketoglutarate levels.^20^ In the brain, NAAG is the most abundant peptide transmitter and acts as a neuromodulator of glutamatergic synapses. NAAG can activate the presynaptic metabotropic glutamate receptor 3 (mGluR3), which inhibits the release of Glu.^34^ From this point of view, the depletion of NAAG during STF may be beneficial to the presynaptic release of Glu. BCAAs and ArAAs, both large neutral amino acids, compete for transport across the blood–brain barrier via the LAT1 AA transporter.^35^ Because ArAAs are precursors of catecholamine neurotransmitters, the homeostasis of BCAAs and ArAAs can affect the production and signaling of neurotransmitters.^29^ A study has indicated that the ratio of BCAAs/non-BCAAs may influence central and peripheral appetite signaling by reducing the synaptic release of 5-hydroxytryptamine from the serotonergic neurons.^35^ By integrating metabolomics and transcriptomics data, our study further suggested that STF might promote dopamine synthesis and dopamine receptor expression in CBL (Supplementary Data 7). These results support previous evidence that CR reduces the loss of dopaminergic neurons in the substantia nigra of mice and increases dopamine receptor expression in the substantia nigra of rats,^36^ both of which are highly relevant to age-related neurodegeneration.

Besides, it has been reported that the favorable effects of CR on the health span are partly mediated by a reduction in core body temperature during CR.^37^ The NO, a gaseous messenger involved in neuronal signaling, inflammation, and central and peripheral thermoregulation, produced through the citrulline–NO cycle, was reported to promote CR-triggered hypothermia.^37^ Our results suggested that STF may facilitate the synthesis of NO in the brain through the citrulline–NO cycle to achieve hypothermia, while producing fumaric acid to compensate for the TCA cycle (Fig. 7). However, NO production is also associated with the generation of reactive nitrogen species, which can suppress the enzymatic activity of methionine synthase, thereby affecting the methionine-Hcy cycle.^38,39^ A marked reduction in the levels of Hcy and methionine was noted during STF. Methionine-derived S-adenosylmethionine is a key methyl donor for histone and DNA methylation.^40^ We found that *Dnmt3a* gene expression increased and *Hnmt* gene expression decreased in the OB during STF, whereas the opposite was observed in CBL (Supplementary Data 7). Studies have demonstrated that CR induces histone post-translational modifications in the liver of mice.^41^ This study provides further evidence that STF might trigger distinct epigenetic changes in different brain regions. Epigenetic changes modulated by CR can lead to metabolic adaptations and improved health coutcomes.^42^

In addition, this study reported for the first time that STF elicits broad elevation in NAEs in both the liver and brain (Supplementary Data 4). NAEs are endogenous lipids and play important roles in cell signaling and cytoprotection.^43^ Anandamide is an endocannabinoid that acts on cannabinoid receptors in the brain to regulate appetite, mood, and pain.^44^ NAEs can activate the same receptors as exogenous cannabinoids, and produce similar effects on appetite and food intake.^43^ Additionally, NAEs can modulate neurotransmitter release and synaptic plasticity in the brain, which may contribute to their effects on mood and cognitive function.^45^ Studies have shown that N-oleoylethanolamine can inhibit glial activation by modulating PPARα and promote motor function recovery after brain ischemia.^46^ N-palmitoylethanolamine has shown neuroprotective properties against oxidative stress in mouse hippocampal neuronal cell lines by increasing phosphorylation of ERK1/2 and Akt and nuclear translocation of pAkt.^43^ Upregulation of NAE-metabolizing enzymes has been reported in human neural progenitor-derived neurons after being exposed to sub-lethal oxidative stress.^47^ A previous study has shown that dietary supplementation with NAE inhibits neuroinflammatory responses and alleviates hippocampal-dependent memory impairment in a LPS murine model.^48^ Taken together, the elevation of NAEs during STF may play an important role in the regulation of energy homeostasis and brain function. These findings provide new insights into the protective mechanisms and adaptive responses of the brain under fasting conditions.

The present study demonstrates that various brain regions have unique metabolic signatures that enable them to fulfill their respective functions. STF can trigger region-specific metabolic changes in different brain regions and organs. This study provides a comprehensive overview of global metabolic signatures across multiple brain regions; however, this brain metabolome atlas could potentially be expanded to include more detailed brain regions in future research. It is important to note that this study only used female mice in the early adulthood stage; therefore, metabolic differences in response to STF among individuals of different ages, hormonal changes, and sexes could not be studied. Further research involving males and females in different estrous states and ages is required to validate these findings.

In summary, this study unveiled the dynamic metabolic remodeling of the brain in response to STF, which included a shift in metabolic patterns involving lipids, BCAAs, ArAAs, neurotransmitters, the citrulline–NO cycle, and methionine metabolism. These metabolic events may play crucial roles in the regulation of energy metabolism, neurotransmitter signaling, and anti-inflammatory and antioxidant responses, and further exert neuroprotection on the brain under fasting conditions. Although, more research is necessary to confirm these findings in humans, these findings could improve our understanding of the mechanisms and biological processes associated with CR and provide new clues for developing efficient CR-mimetic compounds that ultimately benefit our health.

## Materials and methods

### Animals and STF experiment

All the animals used in this study were purchased from the Animal Center of Dalian Medical University. Animal care and study protocols were approved by the Institutional Animal Care Committee of Dalian Medical University. Adult (12 weeks of age) C57BL/6 female mice were housed in a mouse facility under a 12 h light/dark cycle and randomized into STF and control groups. The room temperature was maintained at 22 ± 1 °C. For the STF experiment, mice were fasted for 3, 6, 12, or 24 h, whereas the control group was fed *ad libitum*. Considering the fluctuation in brain metabolite levels associated with circadian rhythms,^49^ all mice were sacrificed at 10:00 am for subsequent sample collection to ensure consistent baseline levels at each fasting time point. All the mice had free access to water throughout the study period. At the end of each time point of the fasting period, all animals were sacrificed, and the following organs or brain regions were separated and collected: OB, COR, HYT, HIP, CBL, BST, SC, and liver. It is worth mentioning that white matter was not removed during the sampling in order to investigate metabolic changes in the original anatomical structure state. All tissues were immediately frozen and stored at −80 °C until further analysis.

### Metabolite extraction and sample preparation

For GC-MS analysis, 10 mg of wet tissue was sheared and placed in a 2-mL centrifuge tube with a pre-added porcelain ball. Tissue homogenization was performed in ice-cold methanol/water (4/1, v/v) using a mixed grinding apparatus (MM-400, Retsch Technology, Han, Germany). After vortexing for 30 s, the mixture was centrifuged (13,000 rpm, 4 °C, 15 min) to precipitate proteins and tissue residues. Then, 800 μL supernatant was transferred into a fresh tube and lyophilized in a vacuum centrifuge. The residue was dissolved in 50 μL methoxyamine solution (20 mg/mL in pyridine) and incubated in a 37 °C water bath for 1.5 h, followed by a silylation reaction by adding 40 μL *N*-methyl-*N*-(trimethylsilyl)-trifluoroacetamide and incubating at 37 °C for 1 h. After centrifugation, the supernatant was aspirated and used for GC-MS analysis. For lipidomic analysis, the lipids were extracted using a methyl tert-butyl ether (MTBE)-Methanol-H2O extraction system. Briefly, frozen tissue was mixed with 300 μL methanol and homogenized using a mixed grinding apparatus, followed by the addition of 1 mL MTBE. After vortexing for 30 s, 250 μL ultrapure water was added to induce phase separation. After vortexing (30 s) and centrifugation, the upper layer was collected and freeze-dried.

### GC-MS analysis

The GC-MS experiment was performed using a QP 2010Plus GC-MS system equipped with an AOC-20i auto-sampler (Shimadzu, Kyoto, Japan). The system utilized a DB-5 ms fused-silica capillary column (30 m × 250 μm × 0.25 μm, J&W Scientific, Folsom, CA). The interface and ion source temperatures were set to 320 °C and 230 °C, respectively. High-purity helium was used as the carrier gas at a constant linear velocity of 40.0 cm/s. The initial oven temperature was 80 °C for 1 min, ramped to 210 °C at 30 °C/min, increased to 320 °C at 20 °C/min, and maintained for 4 min. An electron ionization source was used, and the ionization voltage was set to 70 eV. The mass scan range was 33–600 *m/z*, and the solvent delay was 2.92 min.

### Lipidomics analysis

A hyphenated LC-MS system equipped with a Nexera LC-40 high performance liquid chromatography (Shimadzu, Kyoto, Japan) and a ZenoTOF 7600 mass spectrometer (AB SCIEX, Foster City, USA) was used for global lipidomic profiling. Lipid separation was performed using an ACQUITY UPLC BEH C8 (1.7 μm, 2.1 × 100 mm) column. Detailed chromatographic and MS conditions are provided in the Supplementary Materials.

### Raw data pre-processing

For the GC-MS analysis, the full scan mode was employed to acquire the metabolic profiles. The full scan data of QC samples were converted into a netCDF file using a Shimadzu Postrun workstation. The netCDF file was analyzed using ChromaTOF 4.43 software (Leco Co., USA) to perform baseline correction, noise reduction, peak detection, deconvolution, and library searches (NIST 11, Mainlib, replib, Fiehn, and a homemade metabolite database). The retention index (RI) was calculated using retention time. Metabolites were identified by comparing both mass spectrum in libraries and RI distance of reference standards in our homemade database, and further confirmed using authentic chemical standards. The similarity score cutoff value was >600 for most compounds, and detailed qualitative information on the identified metabolites is provided in Supplemental Table 1. The identified table containing the retention time and characteristic ion was generated and imported into Postrun Analysis software (Shimadzu, Kyoto, Japan) for peak finding. GC–MS browser software (Shimadzu, Kyoto, Japan) was used to perform batch processing and peak integration. For lipidomics, lipid species were identified based on accurate mass, chromatographic retention, and MS/MS fragmentation patterns using the MS-DIAL (Ver.4.90) software. Detailed qualitative information on the lipids is provided in Supplemental Table 4. The peak area of each lipid species was manually integrated using the SCIEX OS (Ver.2.1.6.59781) software. The raw data were normalized to the total intensity of all detected ions in each sample before further statistical analysis. The original metabolomic data have been deposited in the MetaboLights.^50^

### Statistical analysis

Data quality was assessed by calculating the relative standard deviation of the detected metabolites in QC samples and evaluating the distribution of QC samples on the PCA score plot. Multivariate analyses including PCA and PLS-DA were performed using SIMCA software (version 13.0.0.0., Umetrics AB, Umea, Sweden). The PLS-DA model was evaluated using the relative R2 and Q2. One-way ANOVA was used to identify differences among different time points in each tissue sample. Two-sided independent Student’s *t*-test was applied to determine the DEMs for all comparisons between each time point and controls within each organ or brain region. Metabolites were considered differentially expressed if they had a *p*-value < 0.05. All the DEMs in each organ or brain region were visualized on a heat map using the MeV software package (version 4.8.1). Pathway enrichment analysis was performed utilizing the Pathway Analysis module from MetaboAnalyst 5.0, which integrates enrichment and pathway topology analyses. Bubble diagrams for enrichment analysis were created using R package “ggplot2”. The vertical axis represents the enrichment pathway and the horizontal axis shows the rich factor, which indicates the ratio of the number of DEMs in the indicated metabolic pathway to the number of all metabolites annotated to this pathway. The “Number” in circle size represents the number of DEMs that fall into the indicated pathway. Correlations between the metabolite pairs were calculated using Pearson’s correlation coefficient. Box-and-whisker plots and line graphs were generated using GraphPad Prism 8 software (GraphPad Software Inc., La Jolla, CA, USA).

### Supplementary information

Supplementary Materials

Dataset 1

Dataset 2

Dataset 3

Dataset 4

Dataset 5

Dataset 6

Dataset 7

Dataset 8

## Data Availability

All data in this research were presented within the paper and/or the Supplementary Information. The original metabolomic data have been deposited in the MetaboLights with accession number MTBLS8006 (www.ebi.ac.uk/metabolights/MTBLS8006).
