# Supplementary material for: A metabolome atlas of mouse brain on the global metabolic signature dynamics following short-term fasting
Source: Signal Transduct Target Ther. 2023 Sep 8;8:334. doi: 10.1038/s41392-023-01552-y (PMC10484938; doi:10.1038/s41392-023-01552-y)
Supplement: Supplementary file 1 — Supplementary Materials [file 41392_2023_1552_MOESM1_ESM.docx]

Supplementary Materials for

**A metabolome atlas of mouse brain on the global metabolic signature dynamics following short-term fasting**

Yaping Shao^1*^, Zhenfa Fu^1^, Yanfeng Wang^2^, Zhaofei Yang^1^, Yushan Lin^1^, Song Li^1^, Cheng Cheng^1^, Min Wei^1^, Zheyi Liu^2^, Guowang Xu^2*^ and Weidong Le^1, 3*^

^1^ Liaoning Provincial Key Laboratory for Research on the Pathogenic Mechanisms of Neurological Diseases, The First Affiliated Hospital, Dalian Medical University, 193 Lianhe Road, Dalian, 116021, China.

^2^ CAS Key Laboratory of Separation Science for Analytical Chemistry, Dalian Institute of Chemical Physics, Chinese Academy of Sciences, 457 Zhongshan Road, Dalian, 116023, China.

^3^ Institute of Neurology, Sichuan Academy of Medical Science-Sichuan Provincial Hospital, Medical School of UESTC, Chengdu, 611731, China.

***Correspondence to:** Weidong Le (wdle_sibs@163.com); Guowang Xu (xugw@dicp.ac.cn); Yaping Shao (alanna_s@foxmail.com).

**This PDF file includes:**

Materials and methods

Figures S1 to S16

**Other supplementary materials for this manuscript include the following:**

Supplementary Data 1: (Supplementary Table 1-3)

Supplementary Data 2: (Supplementary Table 4-6)

Supplementary Data 3: (Supplementary Table 7-8)

Supplementary Data 4: (Supplementary Table 9-16)

Supplementary Data 5: (Supplementary Table 17-24)

Supplementary Data 6: (Supplementary Table 25)

Supplementary Data 7: (Supplementary Table 26)

Supplementary Data 8: (Supplementary Table 27-34)

**Materials and Methods**

**Chromatographic and MS conditions of lipidomics analysis**

Lyophilized samples were reconstituted in ACN/IPA/H2O (65:30:5, v/v/v/) containing 5 mM ammonium acetate, and 5 µL of the sample was injected into the LC-MS system in the negative ionization mode, while 3 µL was used for positive ionization mode detection. The mobile phases were 3:2 (v/v) ACN/H2O (10 mM ammonium acetate, phase A) and 9:1 (v/v) IPA/ACN (10 mM ammonium acetate, phase B). The elution gradient initiated with 50% B for 1.5 min, linearly increased to 85% B at 9 min, reached 100% B at 9.1 min, and maintained 1.9 min, then came back to 50% B in 0.1 min and held for 1.9 min for post equilibration. The flow rate was set as 0.3 mL/min. The delivery flow rate was 0.3 mL/min, and the column temperature was 60℃.

The ion spray voltage for MS was set at 5500 V and 4500 V in positive and negative ionization modes, respectively. Ion source gas 1, ion source gas 2, and curtain gas were set at 50, 50, and 35 psi. The interface heater temperature was 500℃. The MS scan range was 150-1800 Da in positive ionization mode and 120-1600 Da in negative ionization mode.


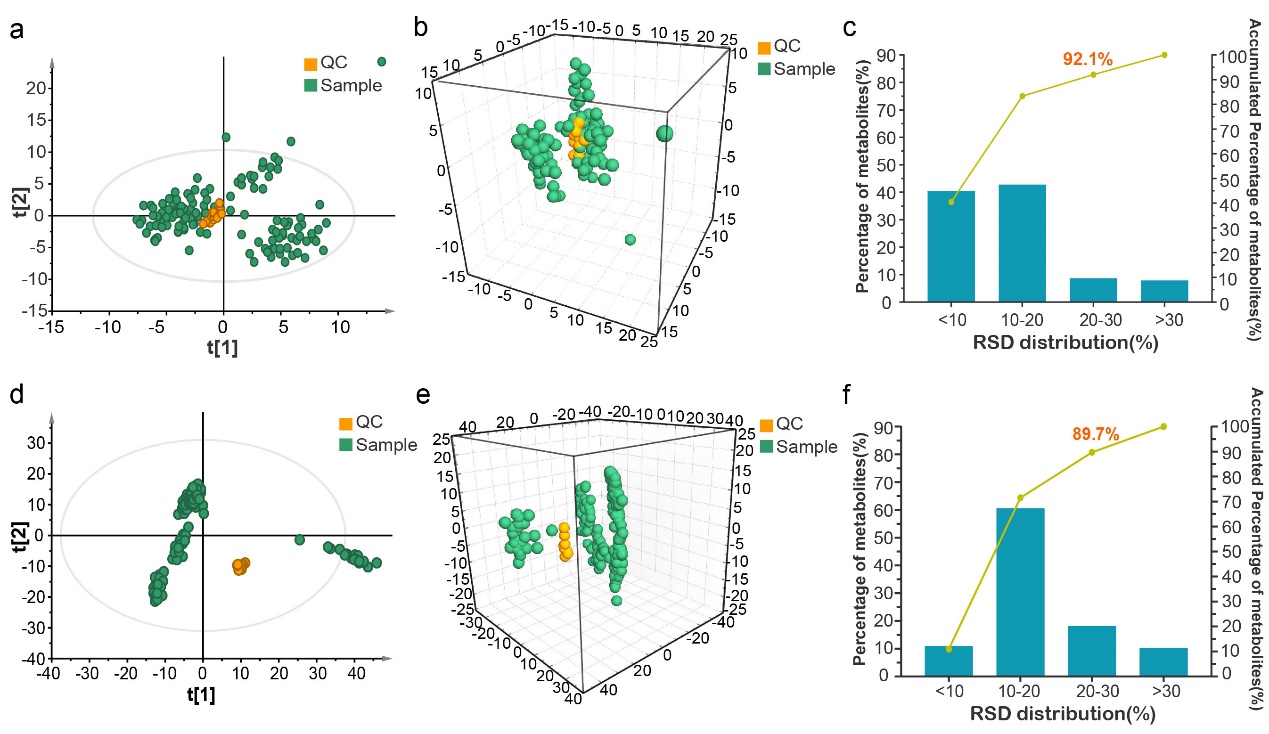


**Figure S1. Quality control of the GC-MS and lipidomics analyses. (a)** Two-dimensional diagram of PCA in GC-MS analysis, with QC samples clustered tightly on the score plot. R2X = 0.306, Q2 = 0.220. **(b)** Three-dimensional diagram of PCA in GC-MS analysis. R2X = 0.382, Q2 = 0.239. **(c)** RSD distribution of detected metabolites in QC samples in GC-MS analysis. **(d)** Two-dimensional diagram of PCA in lipidomics analysis, with QC samples clustered tightly on the score plot. R2X = 0.579, Q2 = 0.571. **(e)** Three-dimensional diagram of PCA in lipidomics analysis. R2X = 0.665, Q2 = 0.654. **(f)** RSD distribution of detected lipid ion features in QC samples in lipidomics analysis.


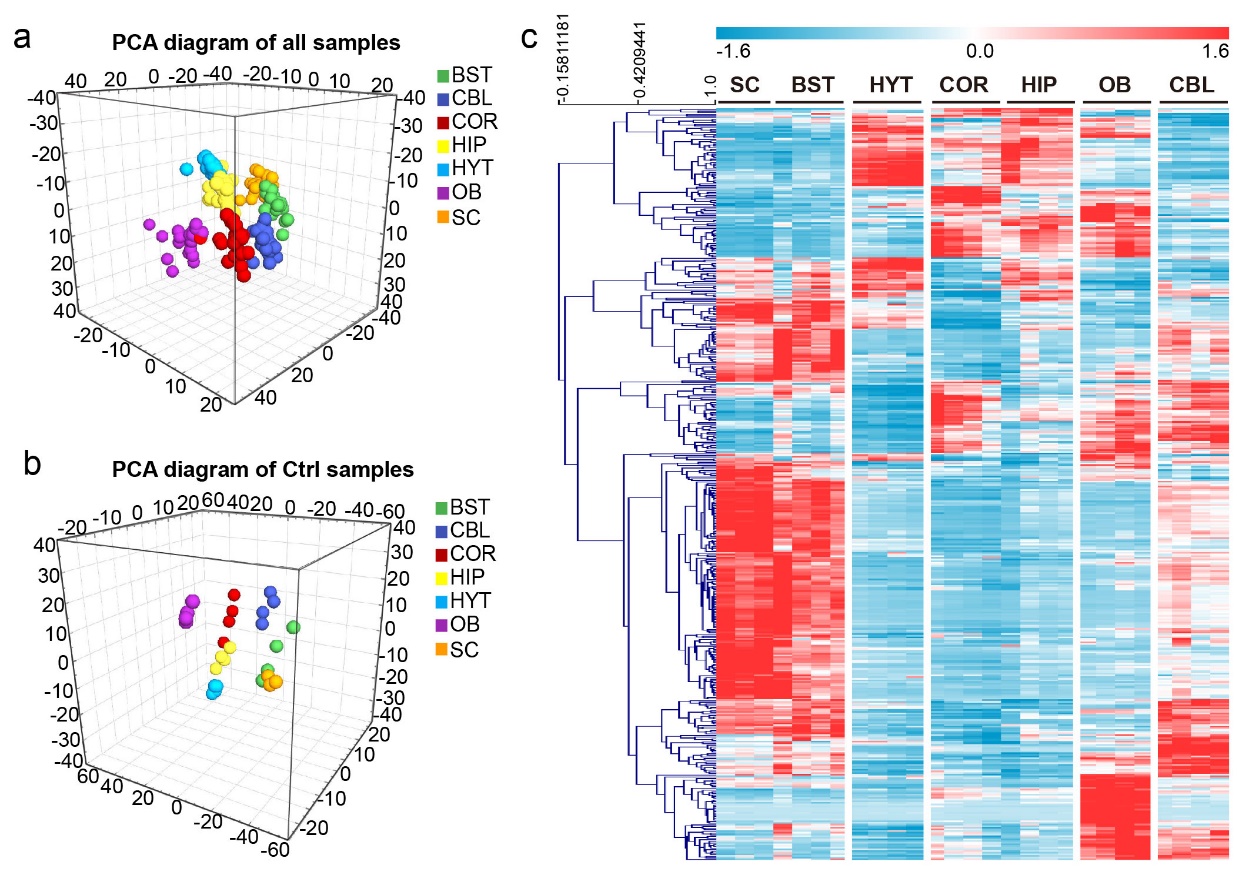


**Figure S2. Metabolic profiles across various brain regions by lipidomics. (a)** Three-dimensional diagram of PCA of brain lipidome from mice fed *ad libitum* or subjected to short-term fasting. R2X = 0.603, Q2 = 0.574. **(b)** Three-dimensional diagram of PCA of brain lipidome from mice fed *ad libitum*. R2X = 0.695, Q2 = 0.588. **(c)** Heat map of the differentially expressed lipids among seven brain regions in mice fed *ad libitum*.


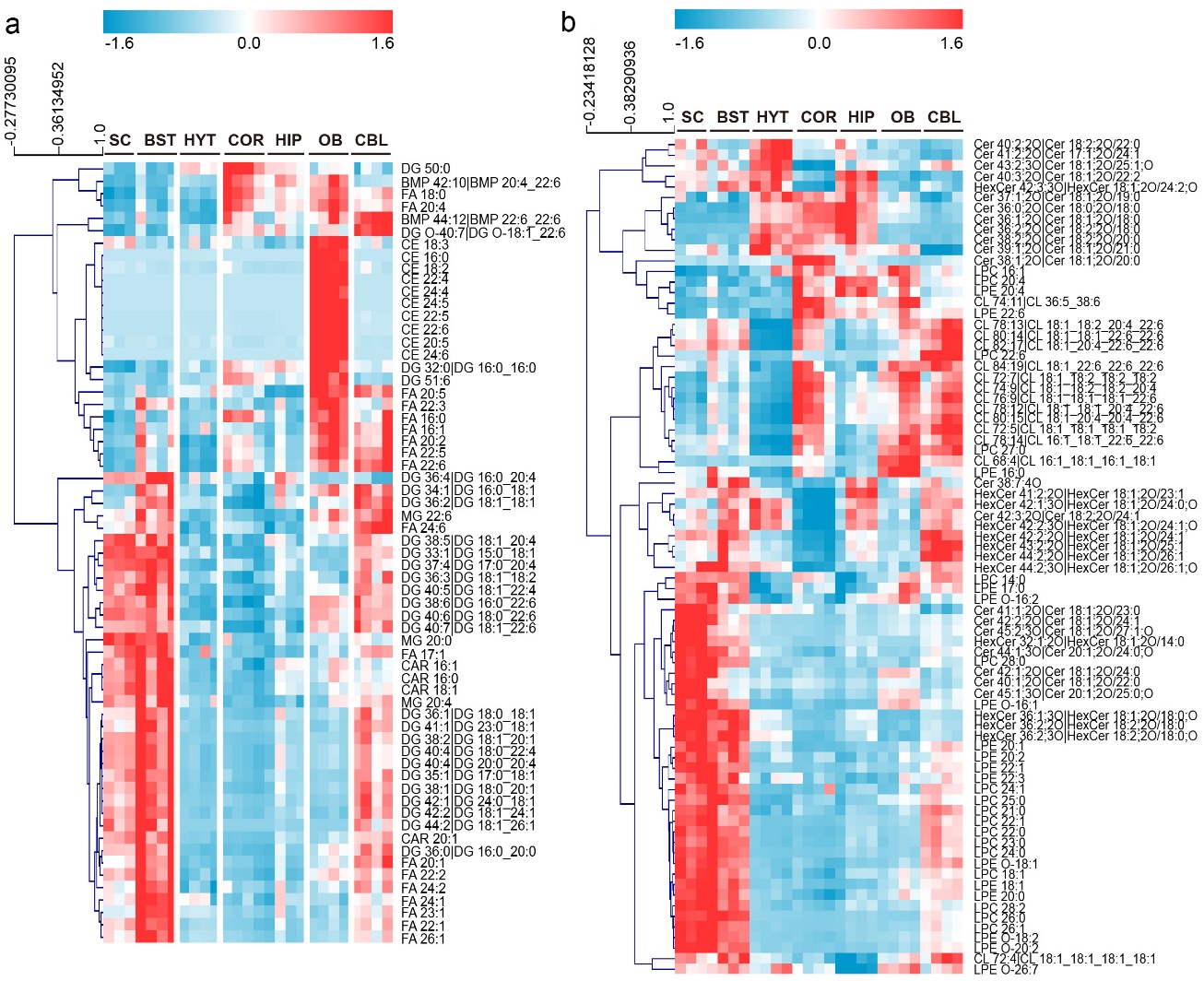


**Figure S3. Heat maps of the differentially expressed lipids among seven brain regions in mice fed *ad libitum*.** The changes of these differential lipids are visualized by class with **(a)** including BMP, CAR, CE, DG and FA and **(b)** including CL, Cer, HexCer, LPC and LPE.


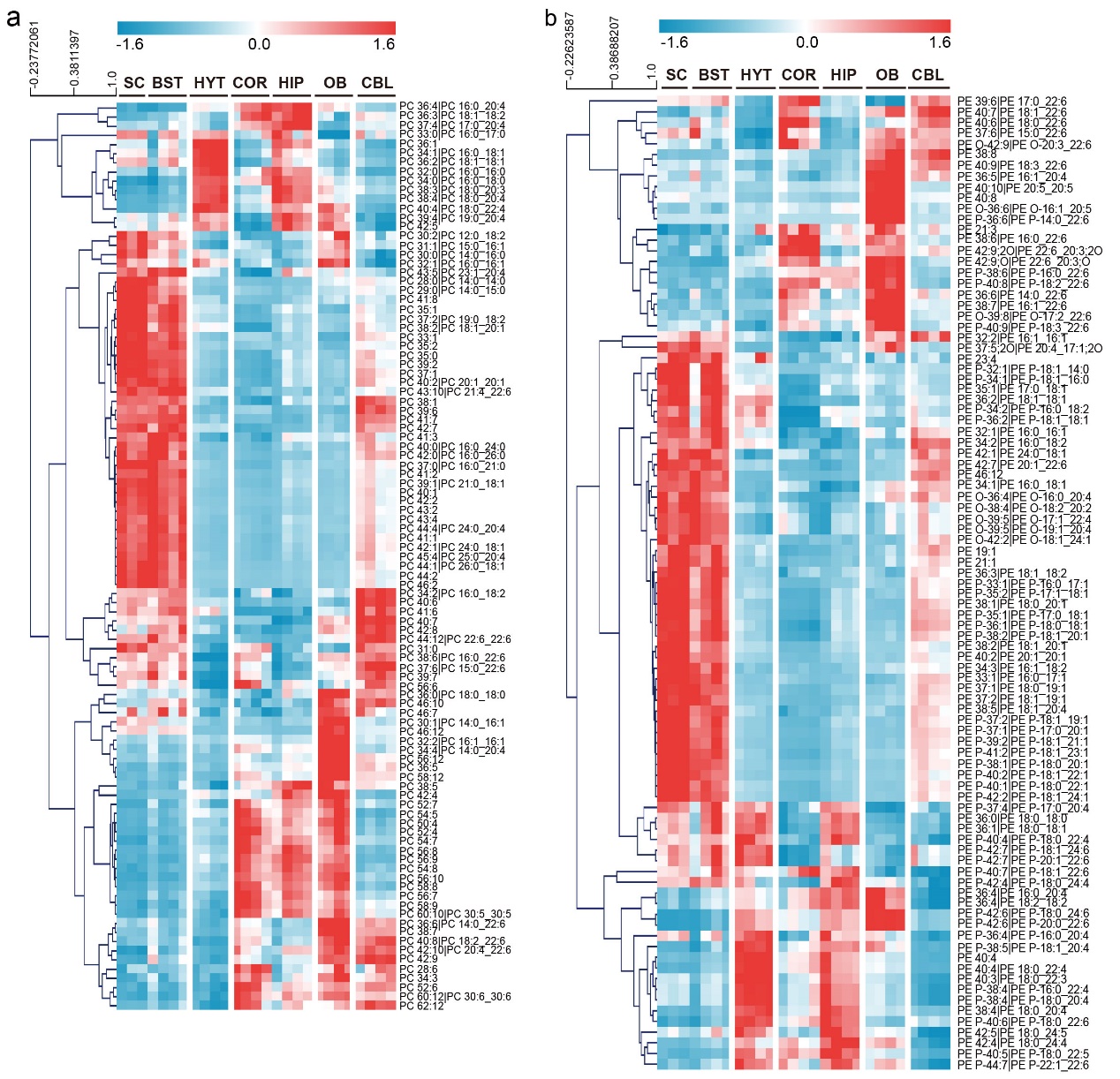


**Figure S4. Heat map of the differentially expressed lipids across seven brain regions in mice fed *ad libitum*.** The changes of these differential lipids are visualized by class with heat map **(a)** PC, **(b)** PE.


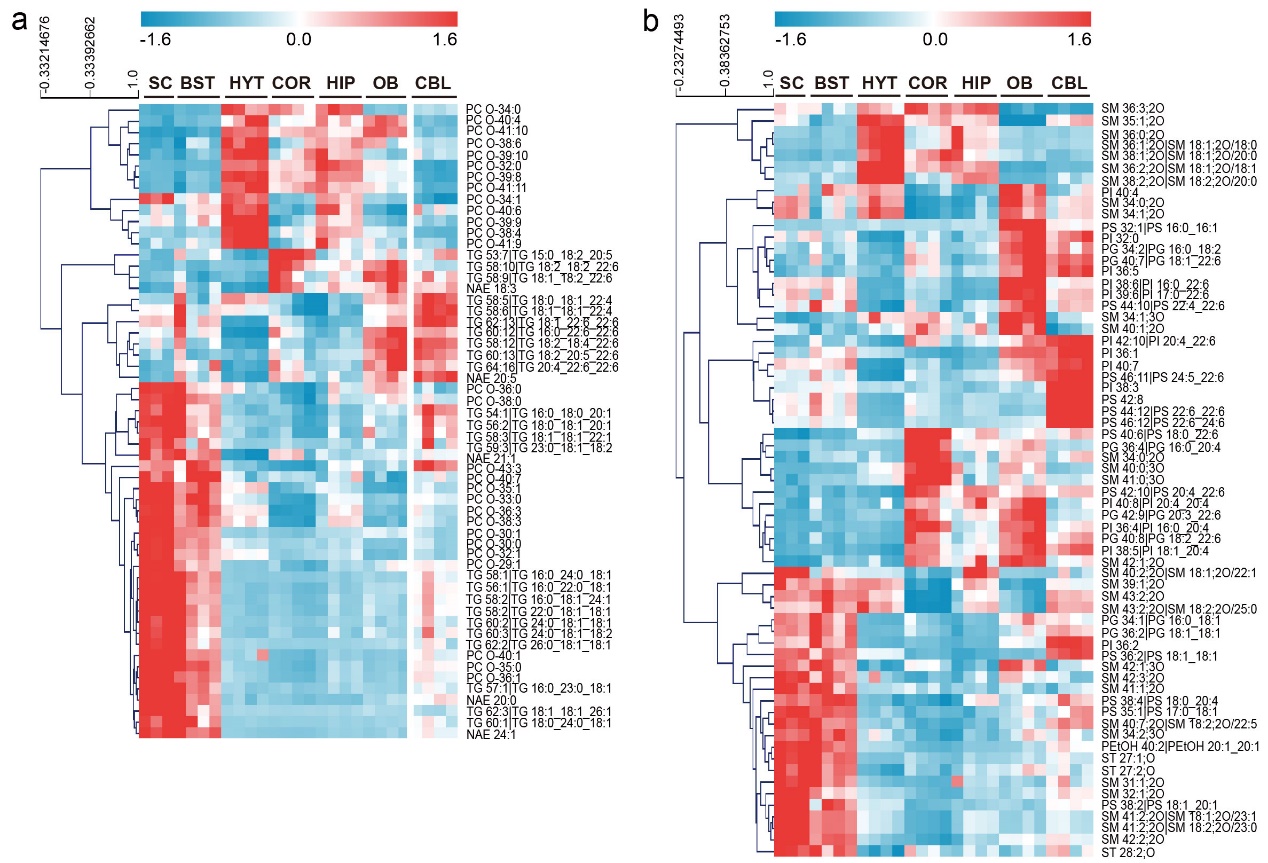


**Figure S5. Heat map of the differentially expressed lipids across seven brain regions in mice fed *ad libitum*.** The changes of these differential lipids are visualized by class with heat map **(a)** PC O-, TG and NAE and **(b)** including SM, ST, PI, PG and PS.


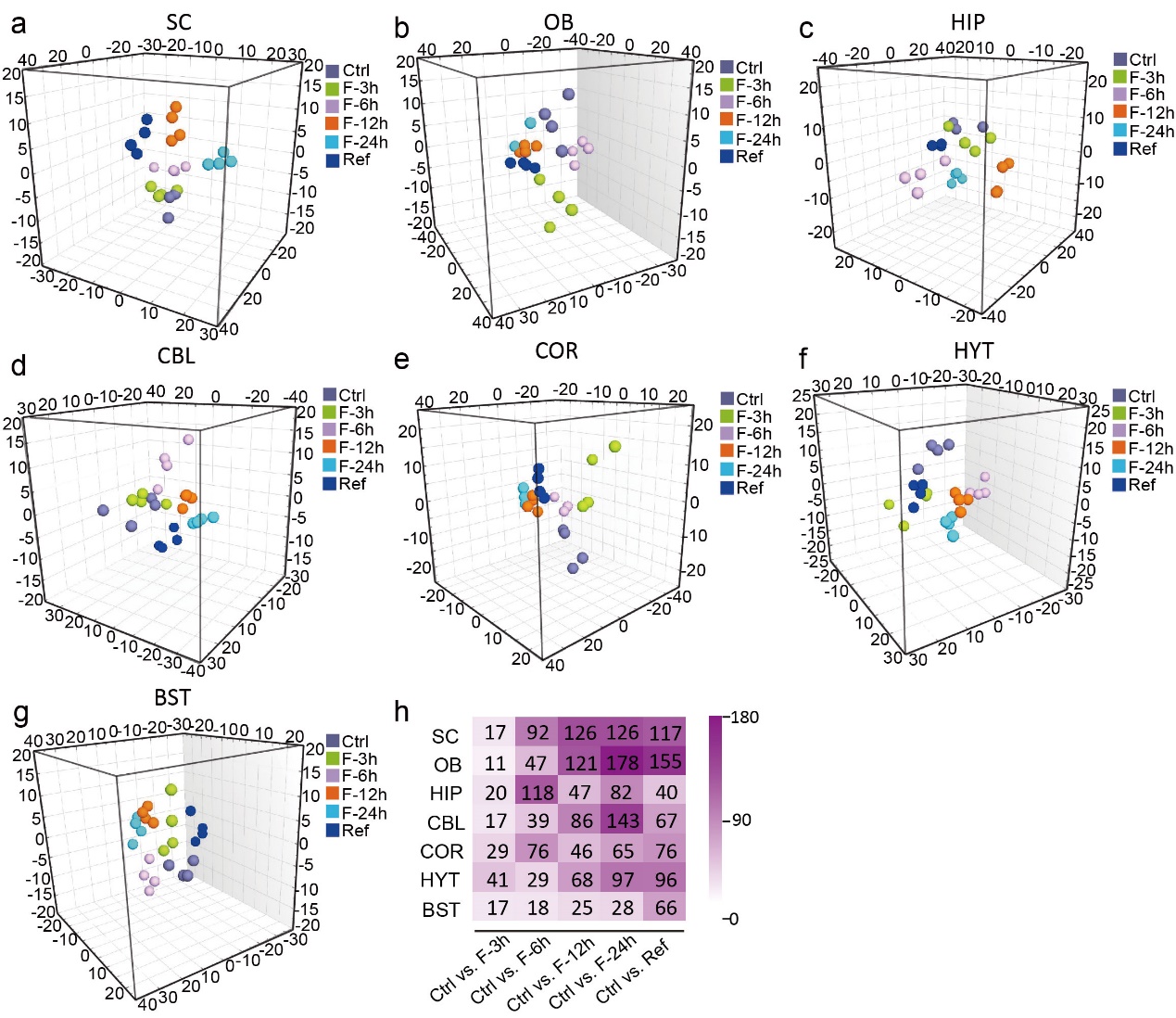


**Figure S6.** **Spatiotemporal effects of short-term fasting on the lipidome in multiple brain regions.** Three-dimensional diagram of OPLS-DA of metabolic response to different periods of short-term fasting in (**a)** SC, R2X = 0.719, R2Y = 0.873, Q2 = 0.239; **(b)** OB, R2X = 0.782, R2Y = 0.828, Q2 = 0.172; **(c)** HIP, R2X = 0.723, R2Y = 0.855, Q2 = 0.137; **(d)** CBL, R2X = 0.738, R2Y = 0.811, Q2 = 0.0975; **(e)** COR, R2X = 0.750, R2Y = 0.833, Q2 = 0.342; **(f)** HYT, R2X = 0.716, R2Y = 0.853, Q2 = 0.316 and **(g)** BST, R2X = 0.693, R2Y = 0.858, Q2 = 0.130. **(h)** Numbers of differential lipids after various periods of short-term fasting in seven brain regions.

**
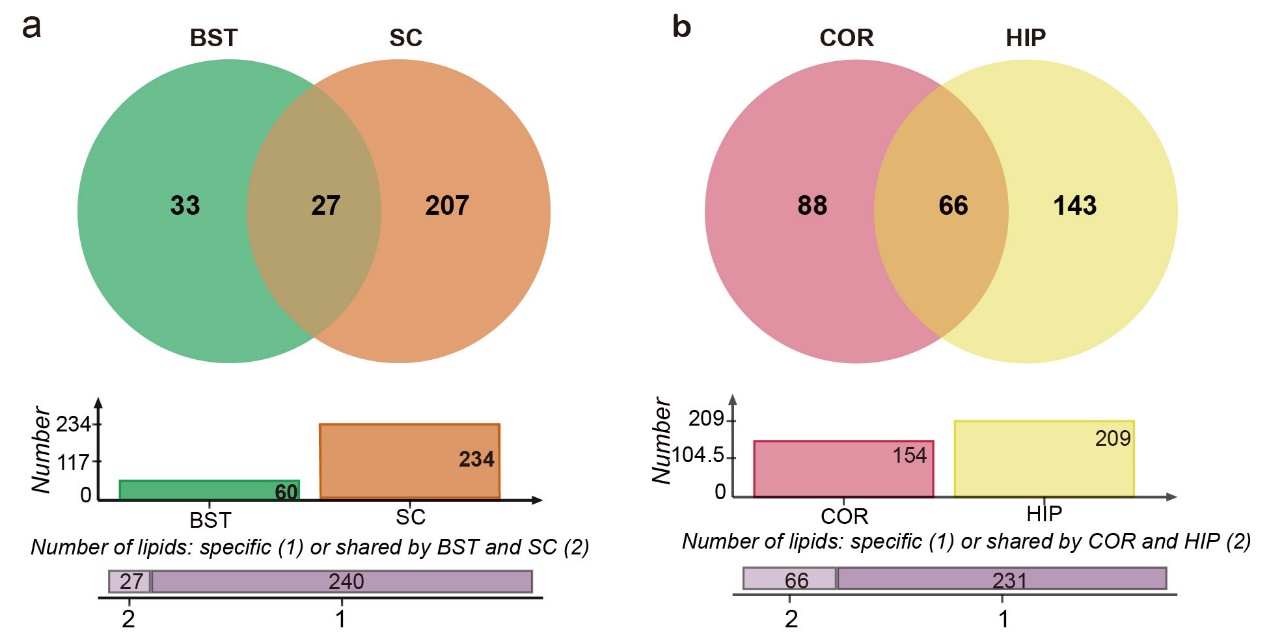
**

**Figure S7.** **Comparison of the lipidome changes after fasting between BST and SC, as well as between COR and HIP.** Venn diagram of the number of differentially expressed lipids after short-term fasting in **(a)** BST and SC, **(b)** COR and HIP. More lipids were found to be changed after STF in SC and HIP, compared with BST and COR, respectively.

**
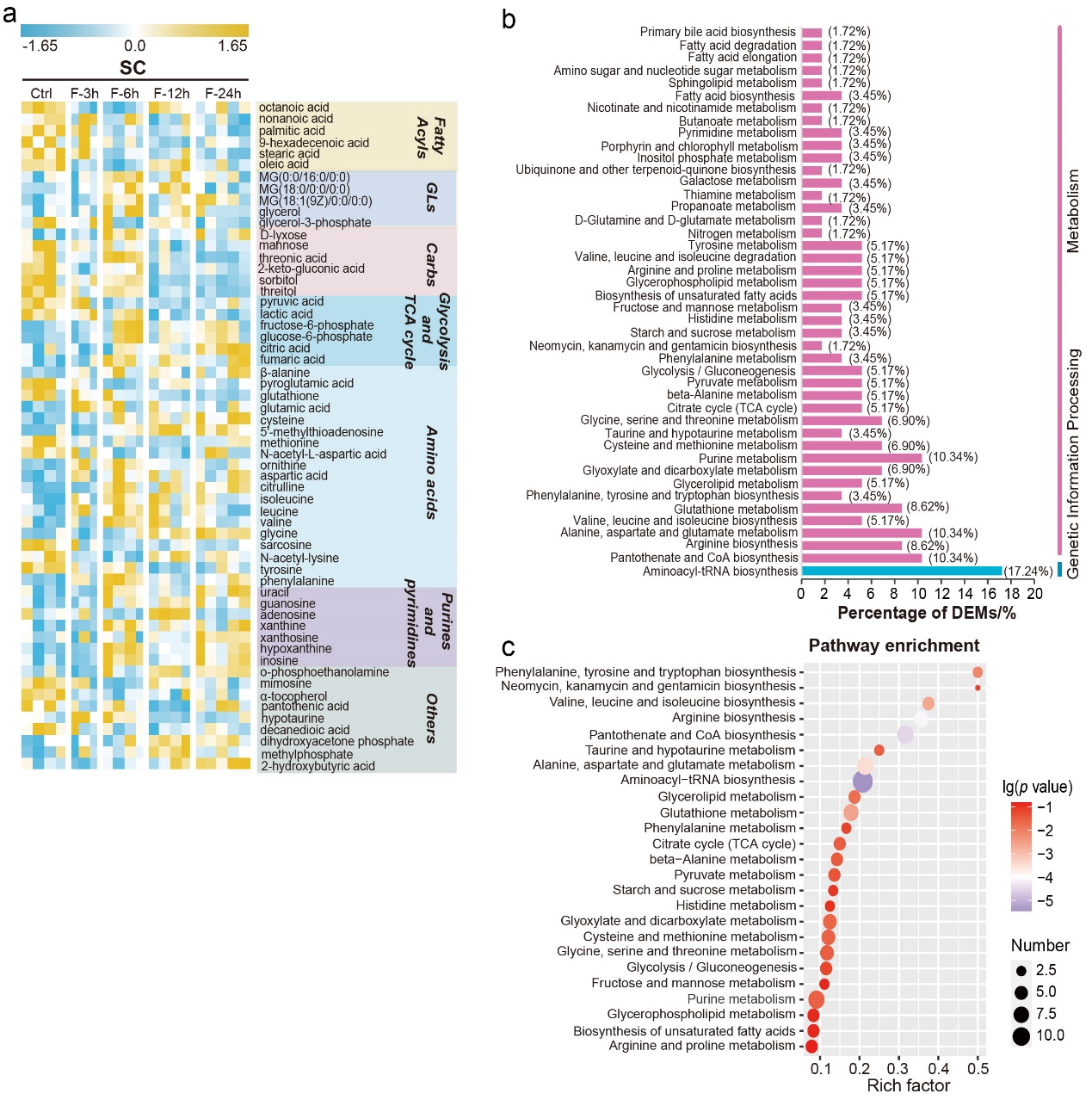
**

**Figure S8.** **Visualization and pathway analysis of differential metabolites responded to fasting in SC. (a)** Heat map of DEMs in SC after short-term fasting. **(b)** Percentage of DEMs in different metabolic pathways in SC. **(c)** Top-25 metabolic pathways that were disturbed after short-term fasting in SC. Pathway enrichment analysis was performed utilizing the Pathway Analysis module from online MetaboAnalyst 5.0 (https://www.metaboanalyst.ca/).

**
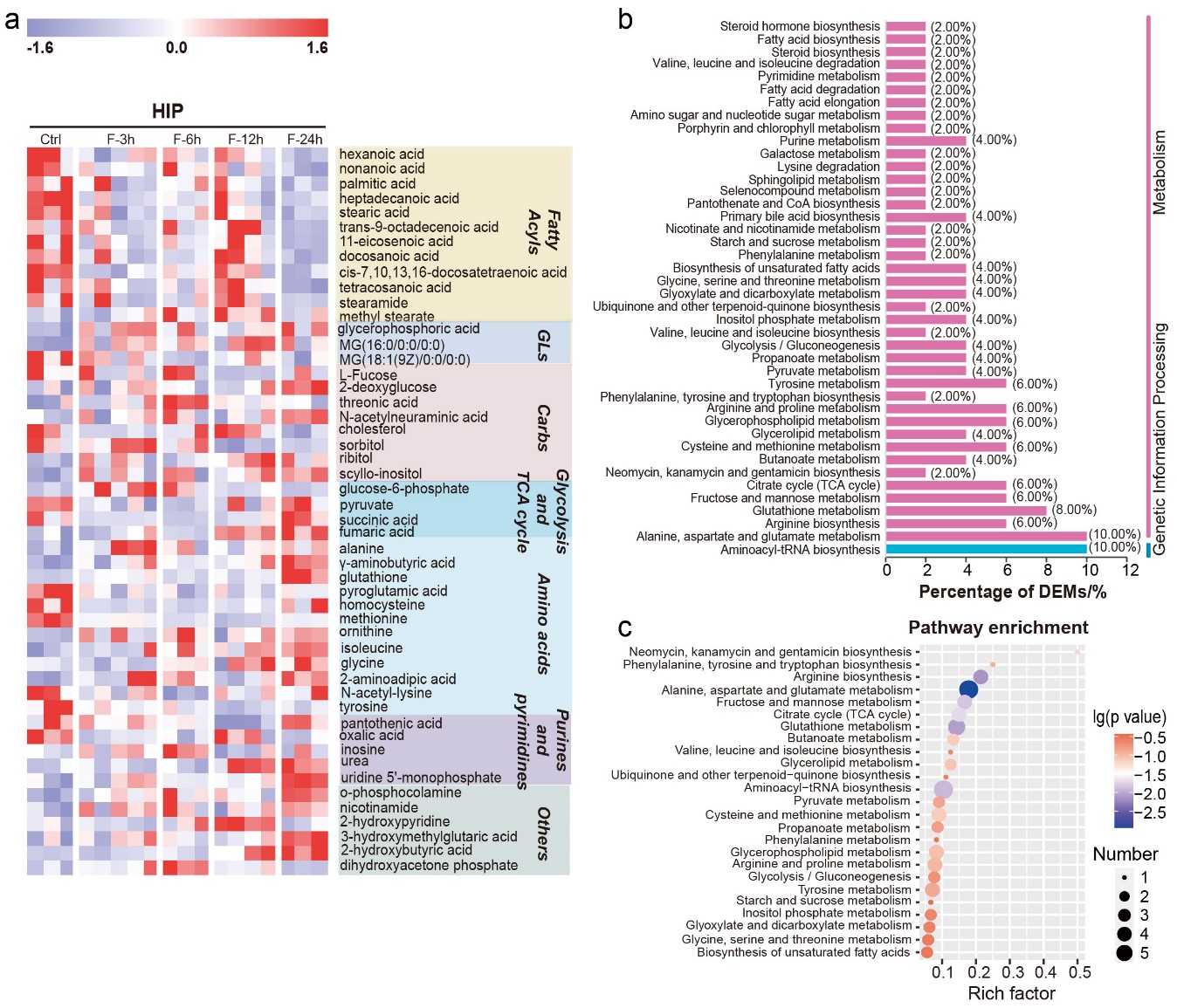
**

**Figure S9.** **Visualization and pathway analysis of differential metabolites responded to fasting in HP. (a)** Heat map of DEMs in HIP after short-term fasting. **(b)** Percentage of DEMs in different metabolic pathways in HIP. **(c)** Top-25 metabolic pathways that were disturbed after short-term fasting in HIP. Pathway enrichment analysis was performed utilizing the Pathway Analysis module from online MetaboAnalyst 5.0 (https://www.metaboanalyst.ca/).

**
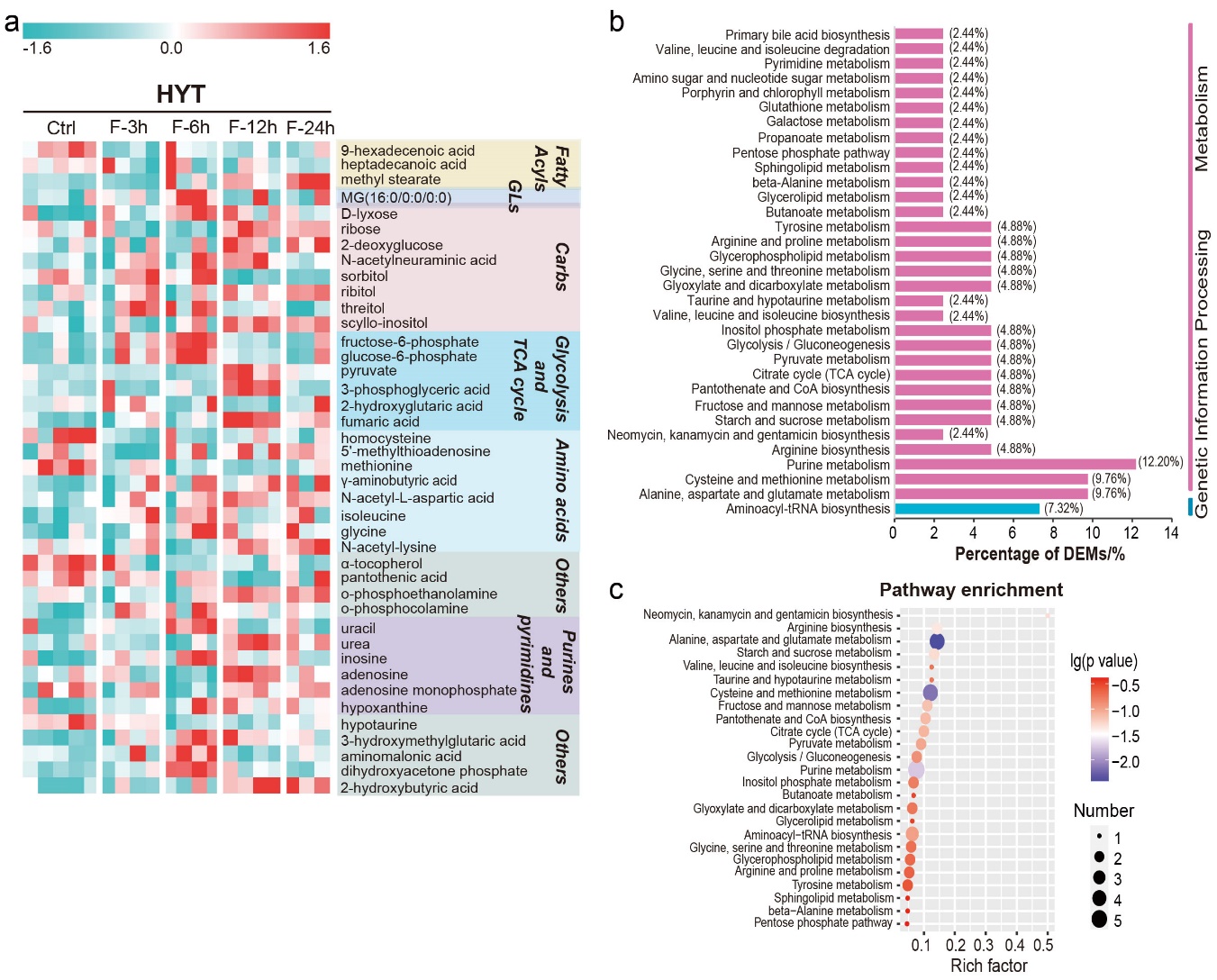
**

**Figure S10.** **Visualization and pathway analysis of differential metabolites responded to fasting in HYT. (a)** Heat map of DEMs in HYT after short-term fasting. **(b)** Percentage of DEMs in different metabolic pathways in HYT. **(c)** Top-25 metabolic pathways that were disturbed after short-term fasting in HYT. Pathway enrichment analysis was performed utilizing the Pathway Analysis module from online MetaboAnalyst 5.0 (https://www.metaboanalyst.ca/).

**
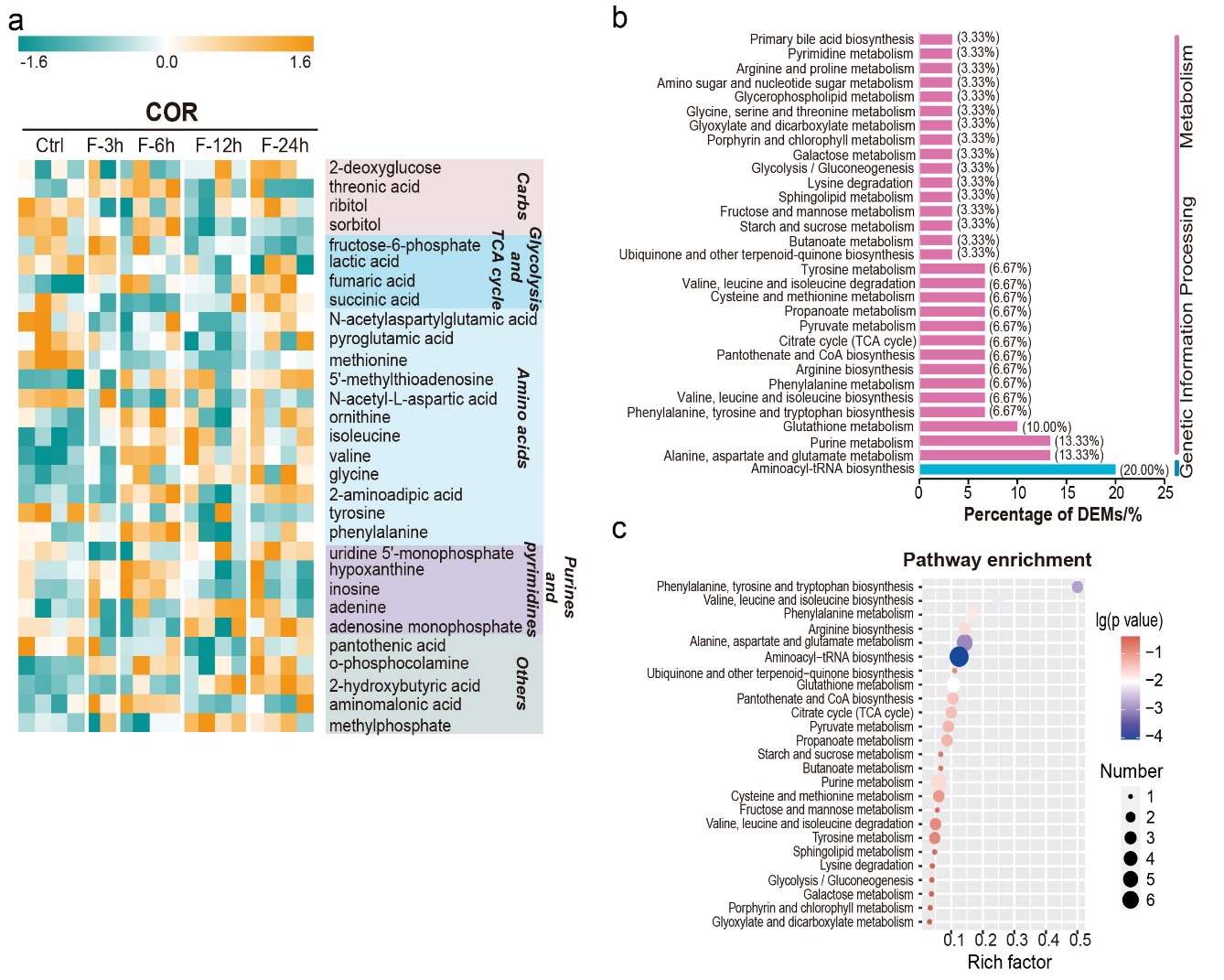
**

**Figure S11.** **Visualization and pathway analysis of differential metabolites responded to fasting in COR. (a)** Heat map of DEMs in COR after short-term fasting. **(b)** Percentage of DEMs in different metabolic pathways in COR. **(c)** Top-25 metabolic pathways that were disturbed after short-term fasting in COR. Pathway enrichment analysis was performed utilizing the Pathway Analysis module from online MetaboAnalyst 5.0 (https://www.metaboanalyst.ca/).

**
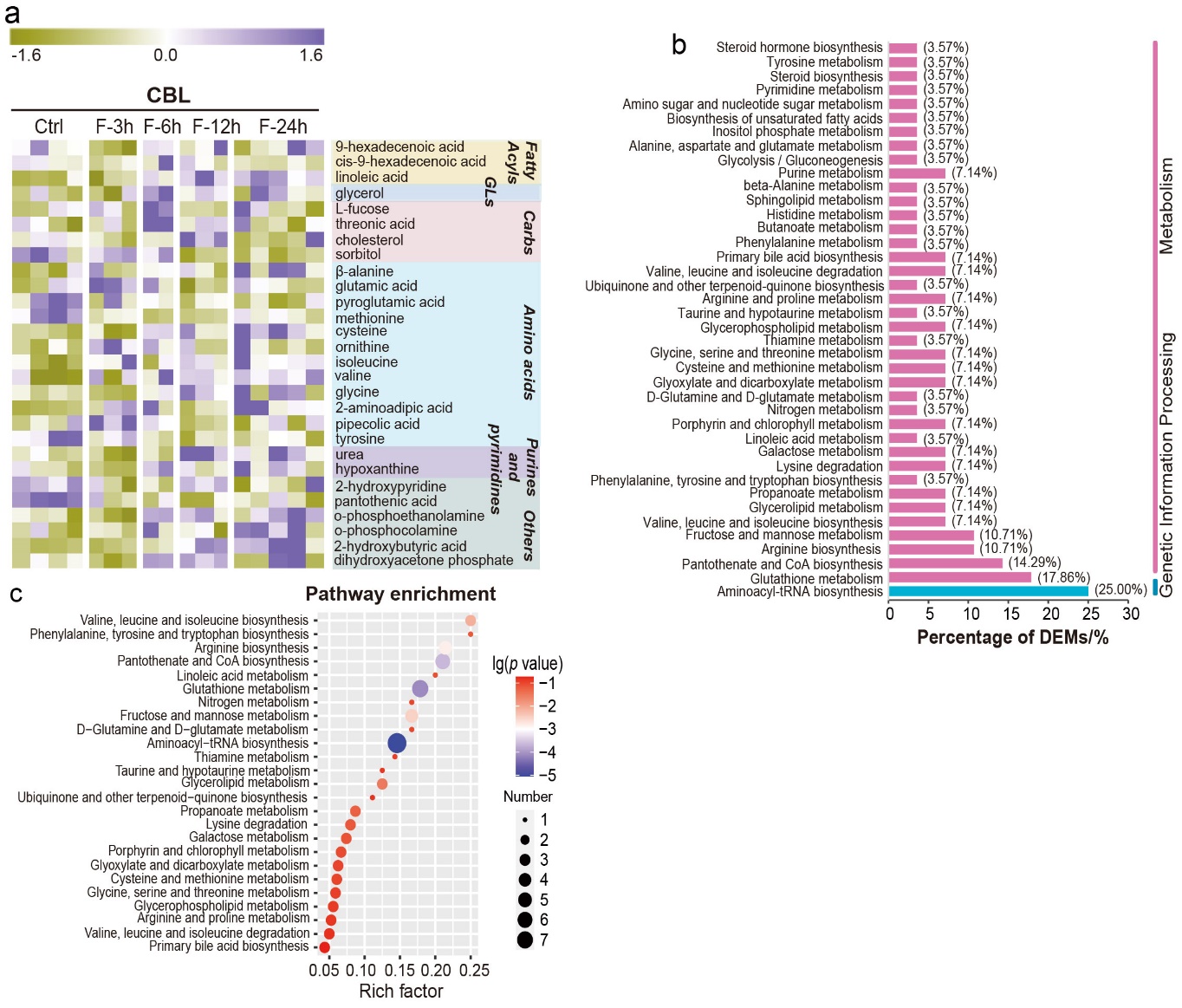
**

**Figure S12.** **Visualization and pathway analysis of differential metabolites responded to fasting in CBL. (a)** Heat map of DEMs in CBL after short-term fasting. **(b)** Percentage of DEMs in different metabolic pathways in CBL. **(c)** Top-25 metabolic pathways that were disturbed after short-term fasting in CBL. Pathway enrichment analysis was performed utilizing the Pathway Analysis module from online MetaboAnalyst 5.0 (https://www.metaboanalyst.ca/).

**
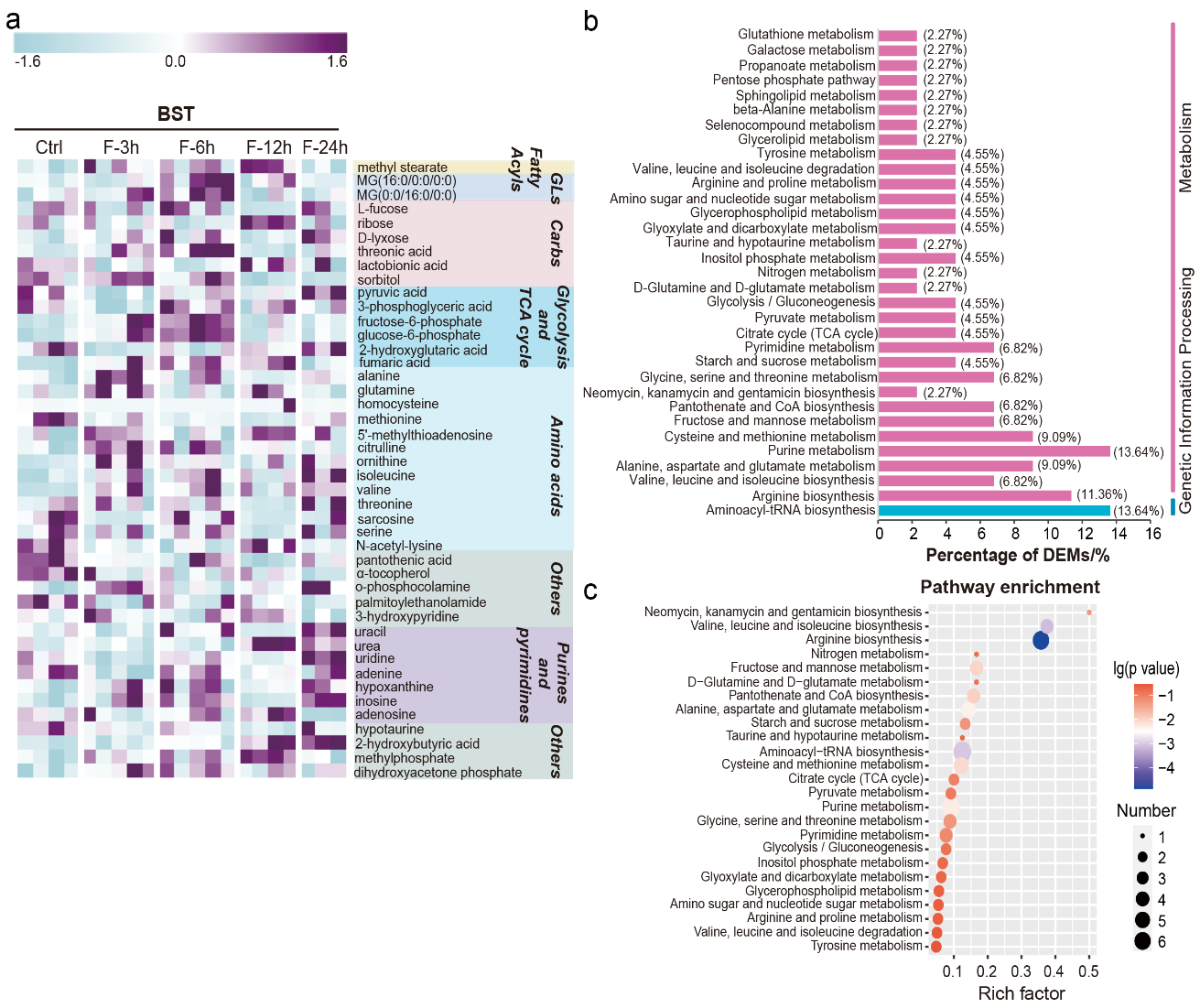
**

**Figure S13.** **Visualization and pathway analysis of differential metabolites responded to fasting in BST. (a)** Heat map of DEMs in BST after short-term fasting. **(b)** Percentage of DEMs in different metabolic pathways in BST. **(c)** Top-25 metabolic pathways that were disturbed after short-term fasting in BST. Pathway enrichment analysis was performed utilizing the Pathway Analysis module from online MetaboAnalyst 5.0 (https://www.metaboanalyst.ca/).

**
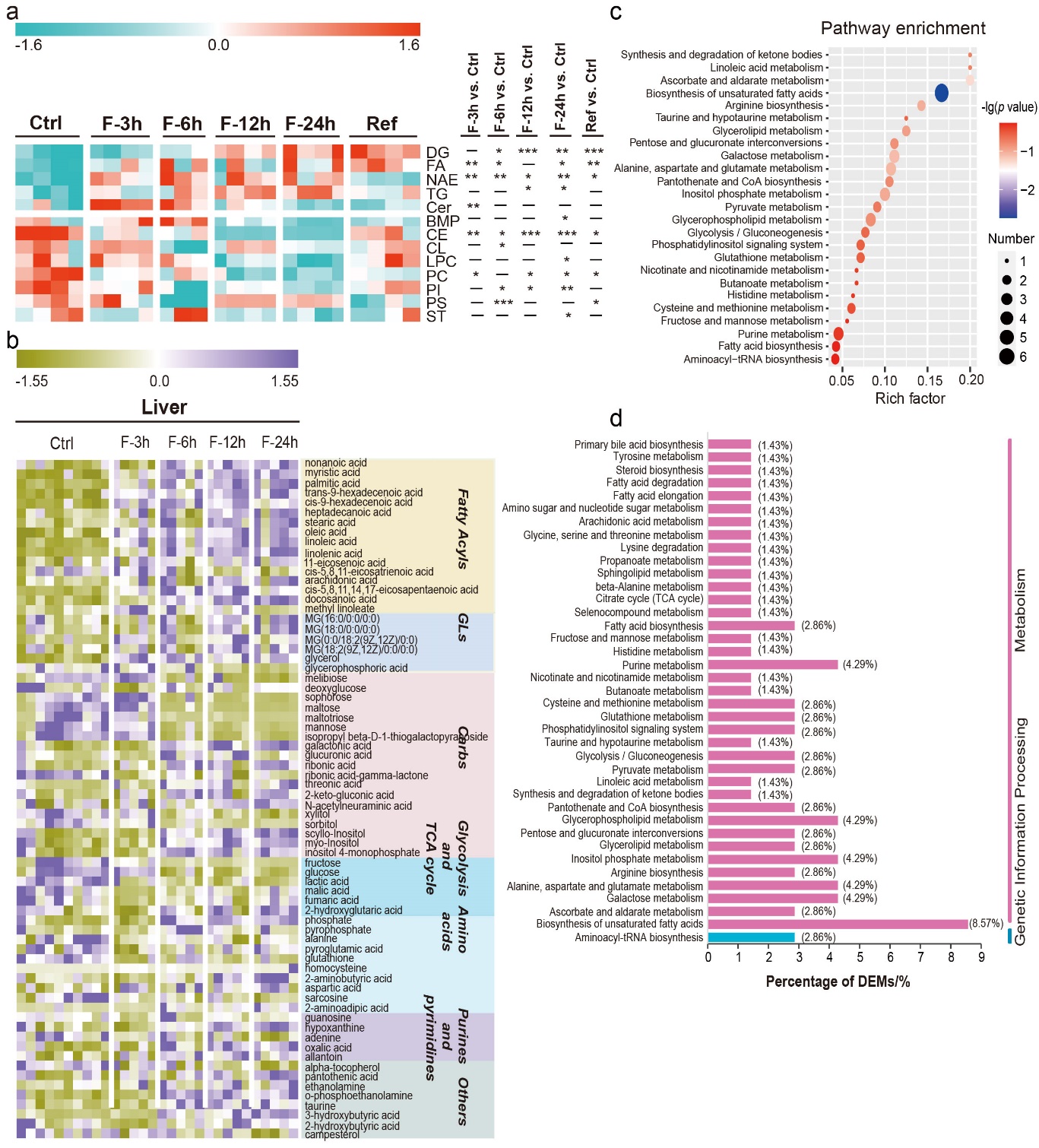
**

**Figure S14.** **Visualization and pathway analysis of differential metabolites responded to fasting in liver. (a)** Alterations of the total amount of lipid classes in liver after short-term fasting and refeeding. * 0.01 < *p* < 0.05, ** 0.001 < *p* < 0.01, *** *p* < 0.001. **(b)** Heat map of DEMs in liver after short-term fasting (GC-MS data). **(c)** Top-25 metabolic pathways that were disturbed after short-term fasting in liver (GC-MS data). Pathway enrichment analysis was performed utilizing the Pathway Analysis module from online MetaboAnalyst 5.0 (https://www.metaboanalyst.ca/). **(d)** Percentage of DEMs in different metabolic pathways in liver (GC-MS data).

**
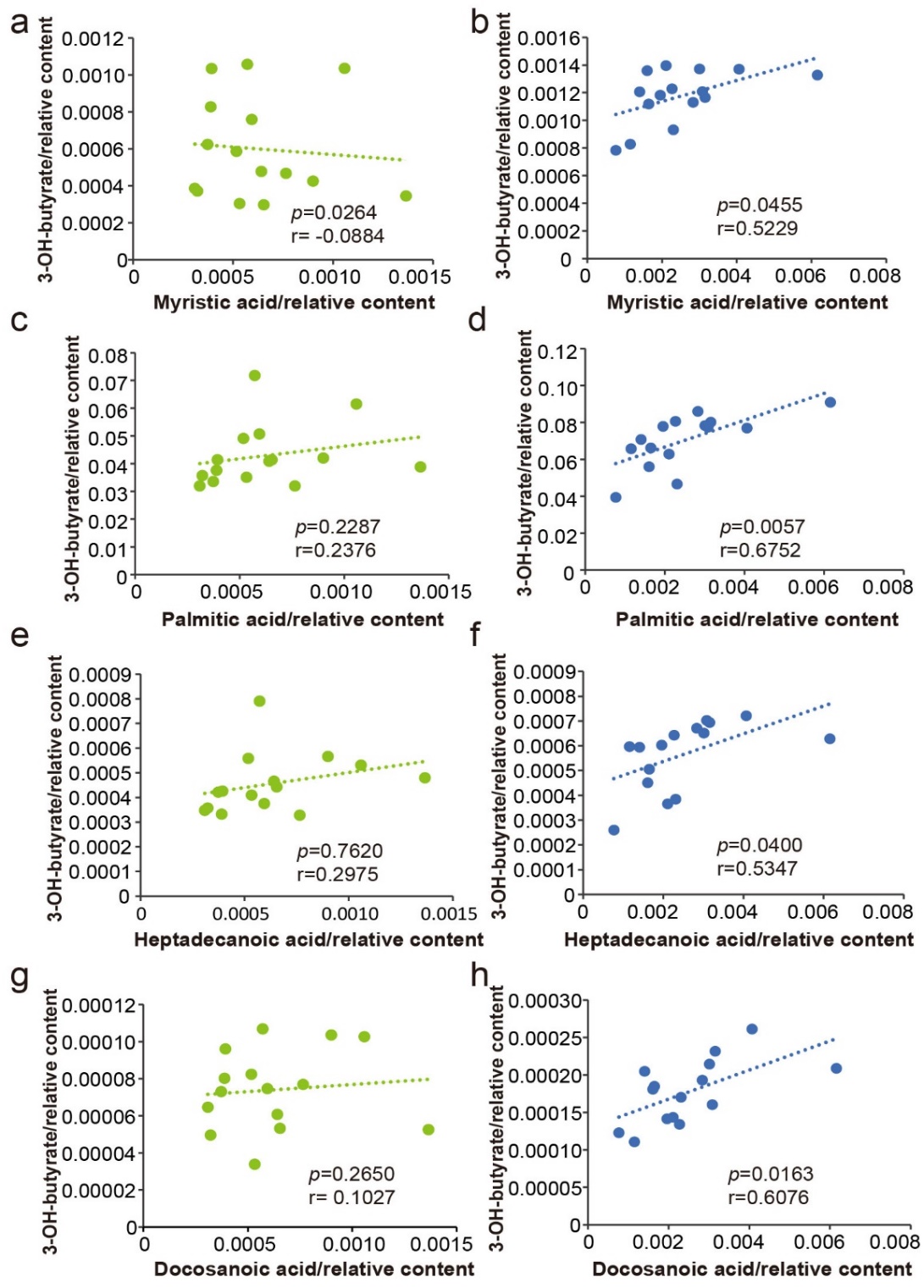
**

**Figure S15. Correlation between** **3-hydroxybutyric acid (3-OH-butyrate) and fatty acids in phase I and phase II in liver. (a,b)** myristic acid; **(c,d)** palmitic acid; **(e,f)** heptadecanoic acid; and **(g,h)** docosanoic acid.

**
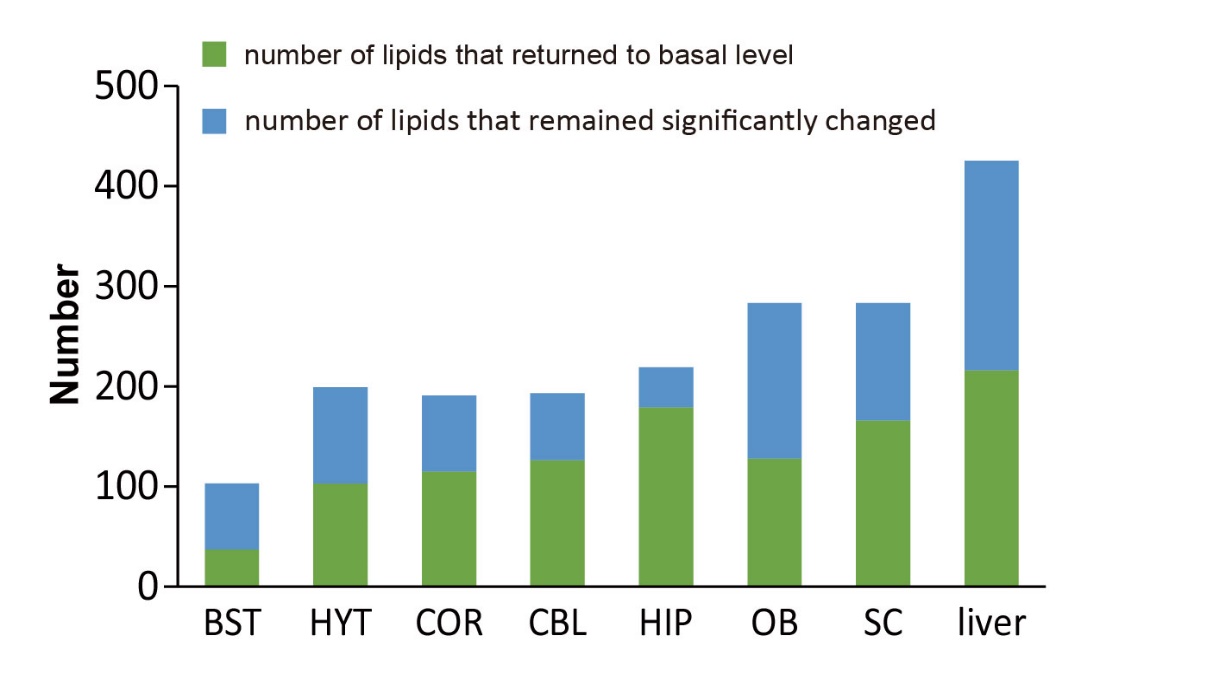
**

**Figure S16.** **Effect of short-term fasting and refeeding on lipidome in brain and liver.**
